# Generation of westerly wind bursts by forcing outside the tropics

**DOI:** 10.1038/s41598-020-79655-7

**Published:** 2021-01-13

**Authors:** Arnold Sullivan, Wenxiu Zhong, Gian Luca Eusebi Borzelli, Tao Geng, Chloe Mackallah, Benjamin Ng, Chi-Cherng Hong, Wenju Cai, An-Yi Huang, Roger Bodman

**Affiliations:** 1 CSIRO Oceans and Atmosphere, Aspendale, Australia; 2 School of Earth, Atmosphere, and Environment, Monash University, Melbourne, Australia; 3 School of Atmospheric Sciences, and Guangdong Province Key Laboratory for Climate Change and Natural Disaster Studies, Sun Yat-sen University, Guangzhou, China; 4 Southern Laboratory of Ocean Science and Engineering (Guangdong, Zhuhai), Zhuhai, China; 5 CERSE-Center for Remote Sensing of the Earth, Rome, Italy; 6 Centre for Southern Hemisphere Oceans Research (CSHOR), CSIRO Oceans and Atmosphere, Hobart, Australia; 7 Key Laboratory of Physical Oceanography/Institute for Advanced Ocean Studies, Qingdao National Laboratory for Marine Science and Technology, Ocean University of China, , Qingdao, China; 8 Department of Earth and Life, University of Taipei, Taipei, Taiwan, ROC; 9 School of Earth Sciences, University of Melbourne, Melbourne, Australia

**Keywords:** Applied mathematics, Climate sciences

## Abstract

The westerly wind burst (WWB) is an important triggering mechanism of El Niño and typically occurs in the western Pacific Ocean. The Fourier spectrum of the wind field over the western tropical Pacific is characterised by a large variety of peaks distributed from intra-seasonal to decadal time scales, suggesting that WWBs could be a result of nonlinear interactions on these time scales. Using a combination of observations and simulations with 15 coupled models from the Coupled Model Intercomparison Project Phase 6 (CMIP6), we demonstrate that the main drivers initiating WWBs are quantifiable physical processes rather than atmospheric stochastic signals. In this study, ensemble empirical mode decomposition (EEMD) from the Holo-Hilbert spectral analysis (HHSA) is used to decompose daily zonal winds over the western equatorial Pacific into seasonal, interannual and decadal components. The seasonal element, with prominent spectral peaks of less than 12 months, is not ENSO related, and we find it to be strongly associated with the East Asian monsoon (EAM) and cross-equatorial flow (CEF) over the Australian monsoon region. The CEF is directly related to the intensity of the Australian subtropical ridge (STR-I). Both the EAM and CEF are essential sources of these high-frequency winds over the western Pacific. In contrast, the interannual wind component is closely related to El Niño occurrences and usually peaks approximately two months prior to a typical El Niño event. Finally, the decadal element merely represents a long-term trend and thus has little to no relation to El Niño. We identified EAM- and CEF-induced westerly wind anomalies in December–January–February (DJF) and September–October–November (SON). However, these anomalies fade in March–April–May (MAM), potentially undermining the usual absence of WWBs in the boreal spring. Similar results are found in CMIP6 historical scenario data.

## Introduction

Westerly wind bursts (WWBs) are a western and central tropical Pacific phenomenon commonly identified as one of the precursors of El Niño events^1–6^. In 2005, Eisenman et al.^7^ suggested that the occurrence and characteristics of WWBs may depend, to some extent, on the state of El Niño-Southern Oscillation (ENSO) components, implying that ENSO itself modulates the WWBs that are associated with the initial onset of ENSO. Westerly wind bursts are often treated as stochastic atmospheric waves that depend the thermocline and produce downwelling, eastward-travelling equatorial Kelvin waves, which create a favourable condition for the development of ENSO. In the available literature, WWBs are defined in several ways. One definition of WWB is a zonal westerly wind anomaly extending at least 10° in longitude, with an average intensity over the western tropical Pacific higher than 5 m/s and a duration longer than 2 days^2,8–12^. Another commonly adopted definition requires the daily zonal wind anomaly over the western Pacific region (5°S–5°N, 135°E–180°E) to be higher than 0.5 m/s^13^. Previous studies indicated that WWBs are characterised by strong seasonal, as well as interannual, variations^14–18^. Some recent studies^19–22^, focusing on wind variability in the tropical Pacific over time scales between 20 and 100 days, argued that WWBs may be related to the Madden–Julian oscillation (MJO)^3,8,10,12,23,24^. However, Puy et al.^8^ and Fu & Tziperman^25^ found that there is no statistical correlation between the two phenomena. A similar result can be found in Hong et al.^21^. MJO and other high-frequency atmospheric signals are mainly related to the Asian monsoon, and as observed by Capotondi et al.^22^, although they can generate Kelvin waves, these waves have amplitudes that are too small to be considered a predominant ENSO forcing. Therefore, in this study, with reference to the literature, we focus on wind variability over time scales between 100 and 365 days, considering MJO as an atmospheric disturbance, which is not able to force efficient Kelvin waves capable of triggering ENSO events.

Other atmospheric processes may also influence WWBs, including the northerly cold surges of the East Asian monsoon^26^ from the Northern Hemisphere and southerly wind anomalies related to the position of the Australian high-pressure system^27,28^. Hong et al.^21^ and Chen et al.^24^ (hereafter: HC^21,24^) suggested that Arctic cold surges from East Asia in mid-March may partly contribute to the subsequent enhancement of westerly wind anomalies in the equatorial western Pacific; these authors attributed the westerly wind anomalies to two pressure systems straddling the western tropical Pacific, which are closely related to the East Asian and the Australian monsoons^20,27^. The influence of the southerly wind anomalies from the Australian continent, on the other hand, is through transverse circulation by a low-level equatorward flow, which spins off from Australian highs and then merges into the deep convection anomalies over the central Pacific^18^.

The East Asian monsoon and southerly winds from the Australian continent serve as significant but indirect extratropical triggers of ENSO events due to their influence on WWBs. Traditionally, the development of these weather anomalies is understood to be dependent on the strength of the Asian monsoon^29^. At the interannual scale, strong El Niño occurrences follow strong Asian monsoons; the strong anomalous northerlies in boreal winter converge with the strong anomalous southerlies associated with the Australian monsoon to produce westerly anomalies in the western Pacific region. As Eisenman et al.^7^ and Capotondi et al.^22^ addressed, monsoon systems can trigger so-called stochastic atmosphere wind anomalies. In this paper, we investigate (a) whether these wind anomalies have their physical dynamics and (b) whether these WWBs are determined by other climate modes.

Another consideration is easterly wind anomalies, which have rarely been studied previously. Zonal westerlies wind anomaly can reverse becoming easterlies, which can in turn inhibit the development of an El Niño event, as in the case of the 2014 El Niño^30–32^. Although these patchy wind patterns (defined by various criteria) have been taken into consideration in the available literature, their prediction and analysis is non-trivial, owing to the complexity of ocean–atmosphere interactions in the tropical Pacific region. The irregularity of WWBs prevents the long-lead predictability of ENSO events^5,19,33^. Therefore, a better understanding of WWBs could improve ENSO predictability. In this study, we focus on seasonal variability to identify the critical components of WWB dynamics with the intent to improve ENSO predictability and dynamics in relation to WWB.

## Results

### Decomposition of the WWBs from the NCEP reanalysis data

**Figure 1:**
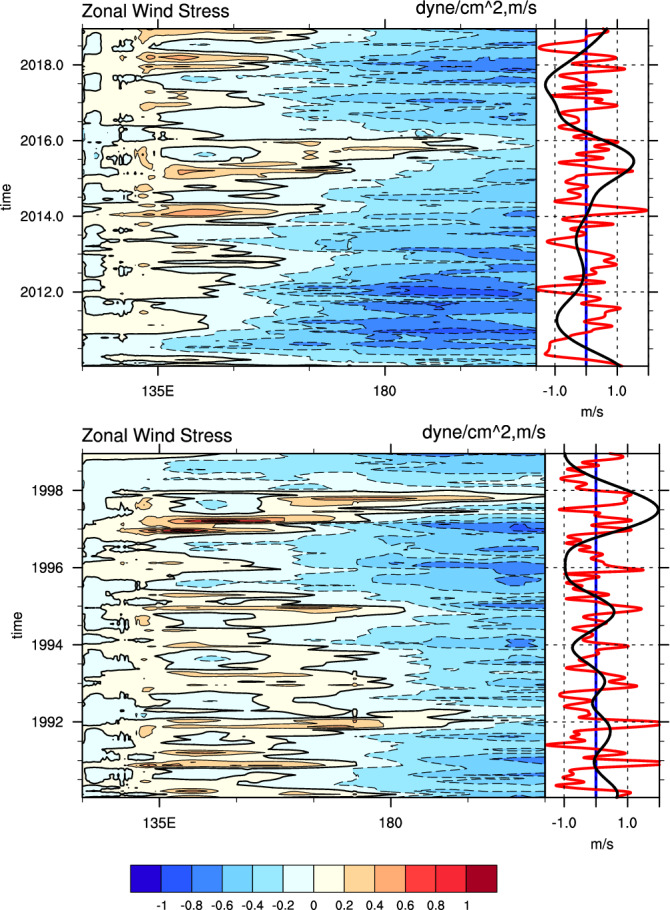
Time-longitude contour plots of the monthly zonal wind stress averaged for 2°S–2°N (left panel; in dyne/cm^2^) interpolated to daily data over two decades covering two extreme ENSO events. The top panel is the 2015/16 case, and the bottom panel is the 1997/98 case. Blue shading represents negative wind stresses, while orange shading represents positive wind stress. The solid contours are positive values and represent westerly winds, and the dashed contours are easterly winds. Right panels are IMFs 6–8 in red and IMFs 9–10 in black curves (units: m/s). Note that the red curves follow each orange peak of the zonal wind stress on the right panel, and the black curves, representing ENSO-related winds, follow the zero lines of the surface zonal wind stress. The black curves indicate the interannual variability, with peaks that follow the ENSO events, and the red curves fluctuate with seasonal variability. Maps were generated using the NCAR Command Language (NCL; https://www.ncl.ucar.edu/), version 6.6.2.

We present two time-longitude contour plots showing daily (interpolated from monthly ocean reanalysis data for plotting purpose to fit to the daily wind on the right-hand panel) zonal wind stresses, averaged over the latitude band 2°S to 2°N, for two extreme ENSO events in 2015/16 and 1997/98 (Fig. 1). Generally, westerly wind stresses occur seasonally, with peaks at the end or early in the year, with a second peak during boreal spring. In some years, the westerlies extend to 150°E, which are commonly identified as ENSO years, and when westerlies overstep the 180°E longitude line, extreme El Niño events take place^5,6^.

The method we applied to derive the intrinsic components is one-dimensional ensemble empirical mode decomposition (EEMD), which is an improved version of empirical mode decomposition (EMD^35^). EEMD is a self- adaptive processing method, decompositing nonlinear and non-stationary signals into several intrinsic mode functions (IMFs). Each of the IMFs represents a pattern embedded in the original signal on different scales (details are in Methods and Supplementary material). IMFs are generally sorted in ascending order of time scale (i.e., the first IMFs represent patterns characterised by shorter time scales). We apply the EEMD to the daily zonal wind components averaged over the region 5°S–5°N, 135°E–180°^13^, and obtain 12 IMFs. IMFs 6–8 are the sum of IMF6 to IMF8,representing seasonal component of WWBs (Fig. 1). IMFs 9–10 is the interannual variance of WWBs. IMFs 1–5 (not shown) are characterised by time scales shorter than scales involved in this study. IMFs 11–12 (not shown) are characterised by scales longer than interannual periods and are therefore considered to be unrelated to the objectives of the research presented here. The IMFs 6–8 tracks both the positive (westerly) and negative (easterly) wind events ever occured. A previous study^32^ argued that the disappearance of the 2014 El Niño event was due to boreal summer easterly wind events, which happened in every year of our analysis (Fig. 1). The variability of these winds is seasonal, with peaks during boreal winter–spring^18^, which we refer to as non-ENSO-related winds. IMFs 9–10 accurately match the zero line of the zonal wind stresses, showing a similar spectrum to ENSO. We refer to these winds as ENSO-related winds.

**Figure 2:**
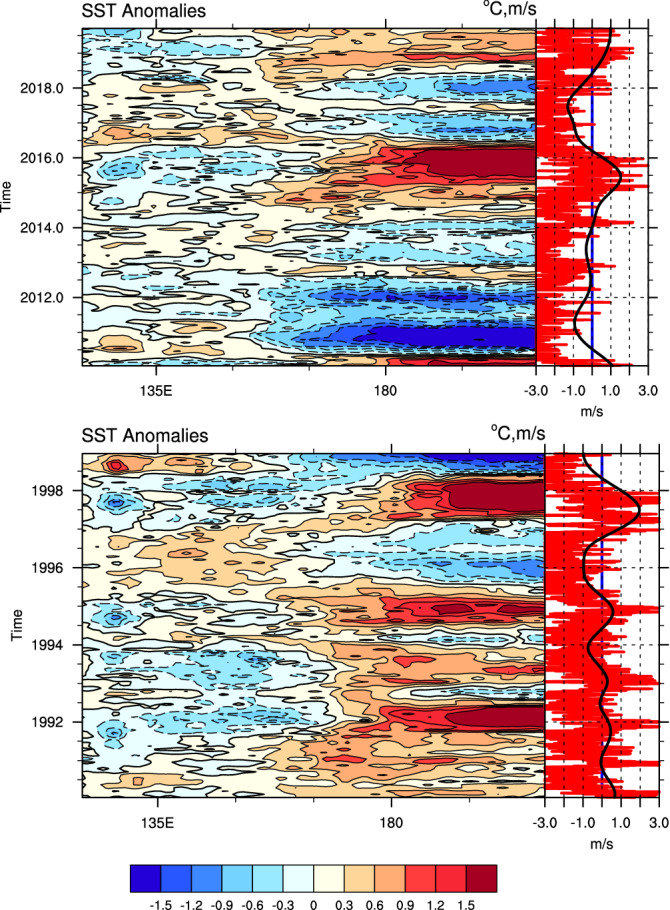
Time-longitude contour plots of the monthly sea surface temperature averaged for 2°S–2°N (left panel; in °C) interpolated to daily data over two decades covered two extreme ENSO events. The top panel is the 2015/16 case, and the bottom panel is the 1997/98 case. The solid red contours are the positive SSTa. The right panels are the same as Fig. 1 but use the traditional method of area averaging over the region 5°S-5°N, 135°E-180°, shown in red curves, and IMFs 9–10 from EEMD are the same as those in the Fig. 1 and shown in black curves (units: m/s). Note that the red curve peaks follow each eastward shift in the red shading of the SSTa in the right panel. Maps were generated using the NCAR Command Language (NCL; https://www.ncl.ucar.edu/), version 6.6.2.

Methods used to define and analyze WWBs are compared in this study. The method proposed by Santoso et al.^13^ gave a patchy pattern similar to Lian et al.^5^ (Figures S1). In Fig. 2, the red curves in the right-hand panels are simply area-averaged daily zonal wind data without EEMD decomposition, while the black curves are IMFs 9–10 from the EEMD analysis (the same as in Fig. 1). Here, the red lines of WWBs is highly correlated with IMFs 9–10, where all peaks are associated with peaks in the ENSO-related wind. IMFs 9–10 is closely related to Niño3.4 indices with a lead correlation coefficient of 0.72 for approximately two months (Figure S2). This ENSO-related wind follows the zero lines of the surface zonal wind stress in Fig. 1. That is, when the negative wind stress retreats towards the eastern Pacific, the ENSO-related wind becomes positive, and vice versa. We then deduce that the ENSO-related wind is the background signal of non-ENSO-related WWBs, previously described as ‘western warm volume-related winds^7,8^, while the seasonal non-ENSO-related winds are the ‘stochastic winds’ described in these papers. To provide a better understanding of the physical mechanisms underlying ENSO generation and predictability, we focus on the seasonal non-ENSO-related zonal wind and attempt to identify the mechanisms that drive this wind to demonstrate that it is not purely stochastic signals.

### East Asian monsoon and cross-equatorial flow are the main factors

**Figure 3:**
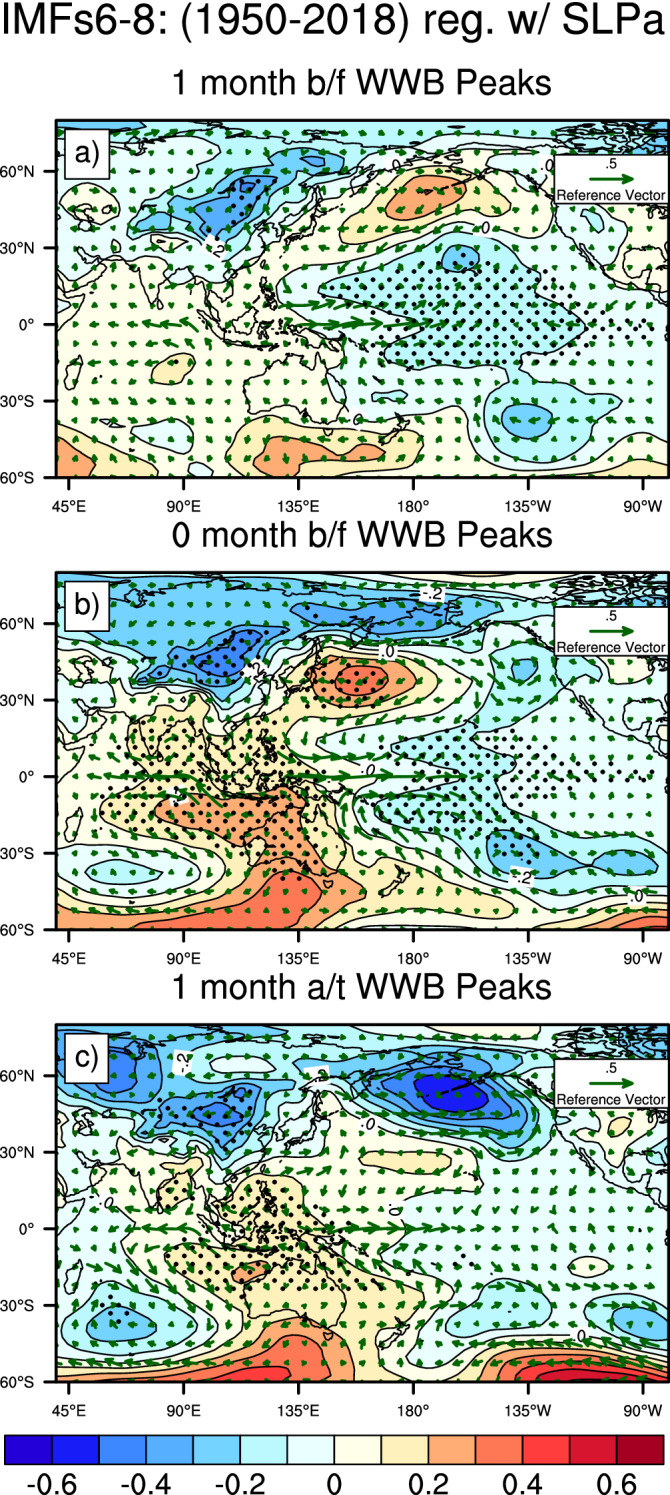
Monthly mean sea level pressure anomalies regressed with IMFs 6–8 from lag 1 months to lead 1 months. The contour interval is 0.1, and the regression coefficients significant at the 99% level according to Student’s t-test are shaded. “1 month a/t WWB Peaks” indicates that IMFs 6–8 leads the SLP anomalies by one month, and the vectors are the winds. Maps were generated using the NCAR Command Language (NCL; https://www.ncl.ucar.edu/), version 6.6.2^57^.

In our EEMD analysis, we decomposed daily zonal wind data into fortnightly, seasonal, annual and decadal signals. All 12 IMF time series are shown in Figure S3, while the corresponding power spectra are presented in Figure S4. The wind components, represented by IMF6 to IMF8, have frequencies of approximately 120, 180, and 360 days, respectively. To focus our investigation on the underlying mechanisms of the seasonal non-ENSO-related winds, we generated regression patterns onto each IMF (Fig. 3 and Figure S5). According to Hong et al.^21^, in the 2015 WWB event, the westerly wind along the equator in the western Pacific region can be triggered by a subtropical high. Chen et al.^23^ suggested that westerly anomalies could be initiated by the Arctic Oscillation (AO) or North Pacific Oscillation (NPO) via the ‘seasonal footprinting’ mechanism^34^. However, it is challenging to evaluate how subtropical highs can drive westerly winds in lead-lag patterns without reliable WWB indices. We, therefore, applied SLPa regression patterns onto the non-ENSO-related wind. Since the dominant frequency of IMFs 6–8 is greater than 90 days and to simplify the calculation, we applied the monthly average on IMFs 6–8. At zero lag time, westerly wind anomalies are associated with two subtropical high systems centred at 40°N, 170°E and 30°S, 135°E (Fig. 3). HC^21,24^ conclude that the Northern Hemisphere subtropical high-pressure system (centred at 170°E and 40°N), which was present in the period 1–5 March 2015, triggered the 2015 westerly wind event. Our analysis of data from 1950–2018 captures this high-pressure system in IMFs 6–8, which then merges with clockwise wind anomalies and induces northerly winds, as was observed from 6 to 10 March 2015. This suggests that both high-pressure systems identified in Fig. 3 initiated WWB events. It was shown that both hemispheres are involved with the commencement of the WWBs. Nevertheless, only the Northern Hemisphere has been explained; further investigation is needed for the Southern Hemisphere.

**Figure 4:**
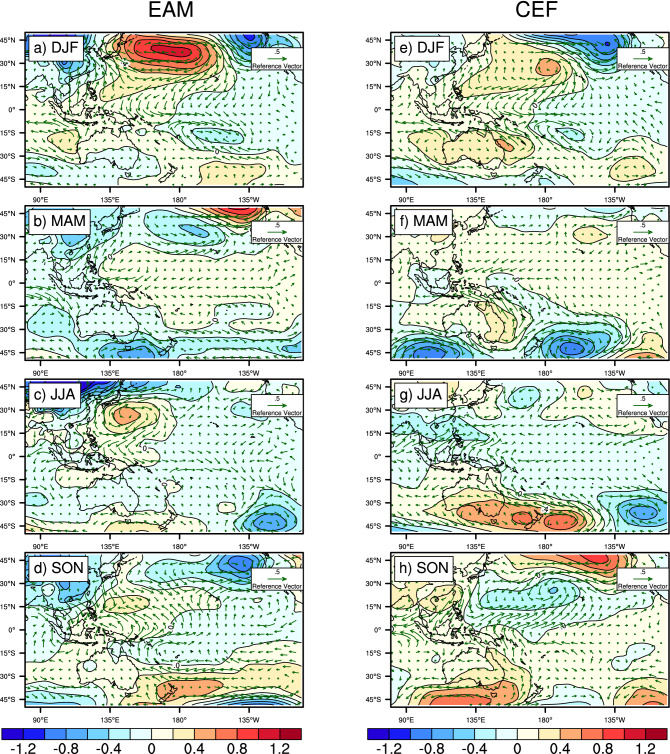
EAS- and CEF-induced sea level pressure anomaly evolution. (**a)** is the seasonal regression patterns of the bandpass-filtered mean sea level pressure anomalies and the winds (as shown in vectors) onto the monthly East Asia monsoon (EAM) index in DJF. (**b–d)** are the same as (**a)** but in MAM, JJA, and SON, respectively. (**e–h)** are the same as (**a–d)** but with cross-equatorial flow (Australian monsoon region). Maps were generated using the NCAR Command Language (NCL; https://www.ncl.ucar.edu/), version 6.6.2^57^.

To illustrate how the Northern and Southern Hemisphere pressure systems influence the western tropical Pacific zonal wind anomaly, we present seasonal regression patterns of monthly SLPa onto the East Asian monsoon (EAM, Fig. 4a–d) and cross-equatorial flow (CEF, Fig. 4e–h) indices. To demonstrate that both the EAM and CEF are the factors involved with the development of the WWBs on the seasonal time scale, we applied bandpass filtering on the mean sea level pressure anomalies before the regression analysis. Both SLPa and the indices EAM and CEF contain seasonal and interannual time scales. Therefore, an 1–14 month bandpass filter is needed to separate the influence from the interannual variability.

Here, we assume that the EAM is induced by the SLPa evolution patterns shown in Fig. 4a-d. Strong westerly anomalies are generated by the extratropical high-pressure system, which is associated with the EAM maximum in the December–January–February (DJF) season. In DJF, the subtropical high induces anticyclone flow along 10°N, and part of the flow moves southward and induces WWBs. Figure 4a shows the retraction of the Eurasian and Pacific highs, causing the easterlies to become dominant in the western Pacific (135°E). Furthermore, the seasonal correlation between the DJF EAM index and IMFs 6–8 of our westerly wind decomposition is 0.29, which is statistically significant, while the correlation with the June–July–August (JJA) season is 0.24 (see Table S1 for a summary of all IMFs 6–8 correlation coefficients). The climatology of the wind pattern during MAM is mainly dominated by the trade wind (not shown) due to the exchange from winter to summer monsoon. The Meiyu system in East Asia halts the pressure system. A negative correlation was found (− 0.41) with the March–April–May (MAM) season EAM index.

To investigate the relationship between southern subtropical highs and westerly wind anomalies, a study in 2014^28^ introduced the Southern Hemisphere booster index of meridional winds averaged over the region 10°S–30°S and 140°E–170°E. Hong and Jin^28^ shows the southerly high merging into the western Pacific and inducing WWBs, with the booster index leading the Niño3.4 index. In an earlier study^29^, a time series of meridionally averaged wind stress anomalies covering the Australian monsoon area (5°S–20°S, 140°E–170°E) also links ENSO development to WWBs^1^, while others^30,36^ have suggested that uneven warming from southern high-pressure systems might induce a more energetic CEF. We find that the more robust southeasterly CEF over the Australian monsoon area merges into the western Pacific, inducing WWBs (Fig. 4). We use the CEF to represent this phenomenon for two main reasons: first, it is more physically related to the tropical variance than the Southern Hemisphere booster, and second, it highlights the role of the Australian monsoon (Fig. 4e). In our analysis, the CEF reaches its highest correlation with our IMFs 6–8 in the September–October-November (SON) season (0.46, see Table S1), with the CEF strongly associated with the intensity and position of the Australian Subtropical ridge (STR-I and STR-P^37–39^) during JJA and SON. The STR-I has shown substantial seasonal and long-term variations that are linked to recent declines in Australian rainfall^40^; however, the STR-I is not directly linked to westerly wind anomalies (the CEF associated with the SLPa evolution patterns shown in Fig. 4g). The extratropical high-pressure system located on the Australian STR in JJA initiates a strong southeasterly wind anomaly in SON. The correlation coefficient between the CEF and STR-I in JJA is 0.64 and in SON is 0.65, while the correlation coefficient between the CEF and the IMFs 6–8 in SON is 0.46. The impacts of the CEF remain until DJF (details in Table S1 and Fig. 4e-h). Meanwhile, no correlation was found between the CEF and IMFs 6–8 in the MAM season.

Both the EAM and CEF can trigger WWBs, albeit in different seasons. The CEF, which related to the intensity and position of the STR, can indirectly impact westerly wind anomalies. Our results provide the missing source of the high-pressure system in the southwest Pacific and introduce a new decomposed westerly wind burst index. Since most WWB definitions state that the westerly wind must last from 5 to 20 days^2,5,9^, our results can explain the long duration of westerly wind anomalies, which is because of the subtropical height system in the Northern and Southern Hemisphere can influence the equatorial wind anomalies in different seasons. However, correlation coefficients between IMFs 6–8 and indices from EAM and CEF are less than 0.3, one might argue whether EAM and CEF are useful for understanding IMFs 6–8. While both coefficients are statistically significant. Thus, the contributions of the EAM and CEF need to be quantified. We used the partial regression between IMFs 6–8 and CEF after removing the EAM in DJF and SON and attempted to achieve a multiple linear regression model that is expressed as ${\mathrm{WWB}}_{\mathrm{fit}} = 0.23 EAM + 0.24 CEF+0.14$, where 0.23 and 0.24 are the partial regressions. The correlation coefficient between IMFs 6–8 and ${\mathrm{WWB}}_{\mathrm{fit}}$ is corr(IMFs 6–8, ${\mathrm{WWB}}_{\mathrm{fit}}$) = 0.38 (p < 0.01) in DJF. The same method was also applied to SON, with a fit of ${\mathrm{WWB}}_{\mathrm{fit}} = 0.21 EAM + 0.34 CEF+0.16$. The correlation coefficient between IMFs 6–8 and ${\mathrm{WWB}}_{\mathrm{fit}}$ is corr(IMFs 6–8, ${\mathrm{WWB}}_{\mathrm{fit}}$) = 0.48 (p < 0.01) in SON. From the multiple linear regression, we find that EAM and CEF do contribute to the WWBs; however, further analysis is needed to identify other factors that play a role in the development of WWBs.

### Robustness from the model simulations

**Figure 5:**
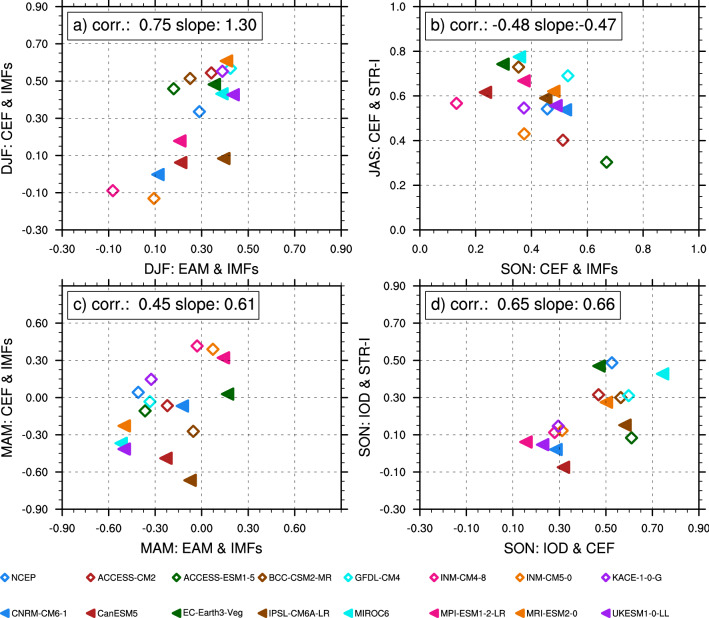
Scatterplot of the correlation coefficient between different indices. (**a)** The correlation between EAM and IMFs 6–8 in December, January, and February (DJF) against the correlation between CEF and IMFs 6–8 in DJF. The correlation in this pair is 0.75 and passes the 99% significance level. The results show a joint relation of EAM and CEF to IMFs 6–8 in DJF. (**b)** The CEF has a significant relationship with IMFs 6–8 in SON, when the CEF is strongly correlated with the intensity of the Australian subtropical ridge (STR-I) in JAS. The correlation between this pair is negative, which means that the strong influence of the STR-I in JAS might reduce the triggering of westerly anomalies by CEF in the following season. (**c)** Both EAM and CEF are negatively correlated to the westerly wind anomaly in MAM. The interaction of both is positively correlated with IMFs 6–8, with a correlation value of 0.45. (**d)** The scatter plot shows that the IOD has a partial impact on both STR-I and CEF in SON; however, there is no significant direct impact on the IMFs 6–8.

We have found that the main triggering mechanism of Pacific westerly wind anomalies is associated with the EAM and CEF. To investigate these triggering mechanisms in more detail, we analysed the simulation data of 15 coupled climate models from the Coupled Model Intercomparison Project Phase 6 (CMIP6^41^), focusing on the historical scenarios. Most of the 15 models can reproduce the negative relationship between EAM and IMFs 6–8 in MAM (Fig. 5c) that we identified in experimental data. Additionally, most of the coupled models mimic the Australian STR intensity and position well (Figure S6) and present deconstructed IMFs with periods of 120, 180, and 360 days in IMF6, IMF7, IMF8, respectively (Figure S7); these values are similar to those of the reanalysis data. We applied the EEMD to the model data and compared the result to that of the NCEP. Most of the models display a robust influence on IMFs 6–8 from both CEF and EAM in DJF (Fig. 5a). Further, models with a higher EAM—IMFs coherence also produce a higher CEF-IMFs coherence, suggesting that both CEF and EAM contribute to the IMFs. We find a strong correlation between the EAM and CEF indices and IMFs 6–8 in most models (see Fig. 5a). The models also generally agreed that IMF6 to IMF8 are the most representative mechanisms of WWBs.

The models included in our analysis that could not well simulate the connection between WWBs and the EAM are INM-CM4-8, INM-CM5-0, MPI-ESM1-2-LR, and EC-Earth3-Veg, which have a weak EAM simulation, as seen in the power spectra of Figure S7 (the power spectra of the EAM, CEF, Niño3.4, IMF6, IMF7, and IMF8 are also shown). While some models show a stronger EAM signal than the NCEP reanalysis on decadal to multidecadal periods, the INM-CM family of models shows very weak simulations of the EAM, CEF, and Niño3.4. Since the EAM and CEF are significant factors in the formation of WWBs and WWBs are a critical factor in the development of ENSO, the weak EAM and CEF seen in the INM-CM models are likely to be the source of problematic ENSO simulations seen in these models. Figure S8 shows the same analysis as Fig. 1 (zonal wind stress time-longitude contour plots, with IMFs 6–8 in red and IMFs 9–10 in black) performed on the historical simulation data from ACCESS-CM2, GFDL-CM4, INM-CM4-8, and UKESM1-0-LL. ACCESS-CM2 and INM-CM4-8 (Figure S8a and S9c) demonstrate more substantial annual periodicity, with IMFs 6–8 peaking in summertime and with less seasonal variation. The IMFs 6–8 signal is particularly pronounced in INM-CM4-8, which, along with ACCESS-CM2, does not reproduce ENSO satisfactorily. In contrast, GFDL-CM4 and UKESM1-0-LL present stronger variability in IMFs 6–8. Indeed, the Niño3.4 power spectra of GFDL-CM4 and UKESM1-0-LL are much closer to those of observational reanalyses (not shown). This analysis provides evidence that realistic ENSO variability cannot be reproduced if the main ENSO related wind components (represented by IMFs 9–10 in our decomposition) are not simulated properly, even if WWBs are captured in the model.

### Why was MJO set aside?

Although the MJO is not the main focus of the research in this study, previous studies have shown that interactions occur between the MJO and WWBs. Seiki and Takayabu ^10^ observed that WWBs were associated with a slowdown of the MJO. Hong et al.^21^ indicated that extratropical highs could affect both the MJO and WWBs, therefore tending to induce the formation of WWBs. Conversely, from our investigation, MJO is only marginally correlated to the seasonal WWB components (Figure S9). We further investigated the nature of WWB IMF5. From a simultaneous correlation analysis between RMM2 (Real-time Multivariate MJO series 2) and WWB IMF5, we find a correlation coefficient of 0.51. However, from a lead and lag analysis, we find that these variables do not impact one another. Between RMM1 and RMM2, the WWB IMFs 6–8 lead 11 days and lag four days, respectively (Figure S9). On the interannual time scale, MJO has a weak correlation with ENSO, which has been identified in previous study^2^. We find that MJO RMM2 leads the ENSO-related wind, IMFs 9–10, by 167 days with a significant correlation, but, the fraction of RMM2 variance that correlates with ENSO (not shown) is too small for further evaluations. For these reasons, the MJO is not included in our research, and further research based on EEMD is required to clarify the factors determining variability in the MJO and its relationship with ENSO.

## Summary

We summarise this study and analysis into two main findings. First, we found that both observations and models display a significant positive correlation between the East Asian monsoon and westerly wind bursts in DJF, with a weak to negative relation in MAM. Our analysis demonstrates that a significant part of the WWB signal is associated with extratropical pressure systems. According to the results presented here, in the western tropical Pacific, strong anomalous northerlies in boreal winter (associated with the EAM) converge with strong anomalous southerlies (related to the Australia monsoon) to produce westerly anomalies in the western Pacific region. We found that the EAM is highly associated with these non-ENSO-related westerly wind anomalies (IMFs 6–8), which are subsequently enhanced in DJF in the East Asia-western Pacific region. Another type of WWB event, induced by cross-equatorial anomalous southeasterly winds along the Australian continent in JJA and SON, further increases the wind stress anomaly in the equatorial western Pacific^24^. Both the EAM and CEF initiate westerly wind anomalies, which may conducive to downwelling Kelvin waves that move warm SSTa from the western Pacific eastward. Another factor considered to be an ENSO precursor is the southerly surge originating in east Australia along 150°E and flowing into the equatorial region. This southerly surge is associated with the CEF over the Australian monsoon region^24^. We also found that the CEF is directly linked to the intensity of the Australian subtropical ridge (STR-I). The STR is well quantified in the east due to a relatively high density of SLP observations in the area, and indices show that the maximum intensity and northerly extent of SLP occur from June to August and that it reaches its minimum intensity from January to February. Nevertheless, in MAM, both factors retreat, and no main climate driver is acting on the western Pacific in these months, other than the background trade wind. In MAM, both factors provide a weak or negative influence on the WWBs in both observations and CMIP6 models. The zonal wind in the western Pacific remains easterly and develops every year. It may explain the easterly event in 2014 that halted the development of an extreme ENSO event.

Our second main finding is that there is no direct link between the Australian STR and westerly wind anomalies in the western Pacific warm pool region. While previous studies^38^ concluded that the STR strengthens and moves poleward under global warming, contributing to reduced rainfall in the cold seasons of southeast Australia, our results (see Table S1) demonstrate a significant correlation between the CEF and IMFs 6–8, rather than between the STR-I and IMFs 6–8 directly. The STR-I is highly correlated to the CEF in JAS (Fig. 5b) but not directly impact to the IMFs 6–8. Then CEF took the place and has a control to the IMFs 6–8 in SON. As a result, the Australian pressure system has an indirect influence on IMFs 6–8 through the CEF. Therefore, in the Australian monsoon region, the CEF plays a significant role in triggering westerly wind anomalies. These results are consistent among the CMIP6 models that we examined. In all 15 models, the CEF and IMFs 6–8 show a strong correlation during the historical period (Fig. 5). We also investigated other climate factors, including the Indian Ocean Dipole (IOD) on the west side of the maritime continent of Australia. Cai et al.^40^ discussed how variability of the STR-I and changes in Austral winter and spring rainfall across southeastern Australia could be better understood through observations of the impacts of remote climate modes, such as ENSO and the IOD. We find that while the IOD is directly associated with the STR-I in SON and with the CEF, the IOD has no immediate effect on westerly wind anomalies during SON (Fig. 5d).

We further decomposed western Pacific zonal winds from the selection of recent CMIP6 historical scenarios. The power spectrum of each mode shows that they are highly independent from one another (see Figure S7). IMFs 6–8 from the models detect each westerly wind anomaly, while IMFs 9–10 represent the background processes that follow the zero line of zonal wind stresses (Figure S8). Note that when the easterlies reach a particular threshold, IMFs 9–10 become negative. Additionally, we note that very few fluctuations can be found in the data from INM-CM4-8, as the model’s Niño3.4 power spectrum illustrates (Figure S7). Since the variation in INM-CM4-8 is too weak (not highlighted), we conclude that the presence of a WWB does not necessarily lead to a well-formed ENSO, as demonstrated in the coupled model results. Simply put, a well-simulated ENSO is not guaranteed without a respectable oceanic response to the interactions between the atmosphere and the ocean.

Finally, we find that the low-frequency ENSO-related wind IMFs 9–10 in our EEMD induces the development and evolution of sea level pressure anomalies in the western Pacific region (see Figure S10) and plays a leading role in the growth of ENSO events. Earlier studies^7,8,22,42^ similarly demonstrate that ENSO development relies on this low-frequency component, as supported by analysis of both observational reanalysis and coupled model data (see Figures S10 and S12). However, a detailed understanding of the mechanisms of this ENSO-related wind requires further research. We conclude that it is unlikely that even a well-simulated seasonal WWB could directly trigger the development of an ENSO event. For example, seasonal westerly wind anomalies from the INMCM4-8 and INMCM5-0 models are markedly higher than those in other models that we analysed; however, only weak Niño3.4 variability was detected in these models. While the primary initiation mechanism of ENSO events is still unclear, the new methodology of decomposing climatological data with EEMD presented in this study provides us with a new perspective on the components of climate phenomena in the Pacific. Our analysis shows that easterly wind anomalies can occur every boreal spring. It will also help to provide a broader understanding of ENSO dynamics and its spring predictability barrier.

## Methods

### Westerly wind burst (WWB) index

To obtain the WWB index, we applied the decomposition method to the daily zonal wind components averaged over the region 5°S–5°N, 135°E–180°^13^ on both observational reanalysis data and model simulation data. Because the target frequencies for this research are based on seasonal signals, the regression analysis uses monthly averaged data from daily data.

### Observational, reanalysis, and CMIP6 model datasets

In this study, we used a few meteorological datasets, including different time scales, to complete this analysis. To retain the daily Niño 3.4, we use the sea surface temperature (SST) data from the NOAA OI SST V2 High Resolution Dataset (NOAA High Resolution SST data provided by the NOAA/OAR/ESRL PSL, Boulder, Colorado, USA, from their website at https://psl.noaa.gov/). For the monthly mean data, for convenience, we used monthly SST data spanning from January 1850 to March 2019, provided by the Hadley Centre Sea Ice and Sea Surface Temperature data set (HadISST1^43^). Daily atmospheric reanalysis data covering the period January 1948 to March 2019 were provided by the NOAA-National Centers for Environmental Prediction (NCEP^44^). Monthly zonal wind stress data were obtained from the Ocean ReAnalysis System 5 (ORAS5)^45^ dataset, which is shown in Fig. 1. However, for the plotting, the zonal wind stress data have been interpolated daily for easy comparison to the IMFs in Fig. 1. For comparison and quality control of NOAA atmospheric data, six-hour ERA-Interim 10 m zonal (U) wind components covering the period January 1979 to March 2019 were used. Due to the limited availability of daily data for the 64-year period of 1950–2014, 15 historical Coupled Model Intercomparison Project Phase 6 (CMIP6^41^) were used in this study: ACCESS-CM2, ACCESS-ESM1-5, BCC-CSM2-MR, GFDL-CM4, INM-CM4-8, INM-CM5-0, KACE-1-0-G, CNRM-CM6-1, CanESM5, EC-Earth3-Veg, IPSL-CM6A-LR, MIROC6, MPI-ESM1-2-LR, MRI-ESM2-0, and UKESM1-0-LL.

### East Asian monsoon (EAM) index and the Australian monsoon-related cross-equatorial flow (CEF)

Following Chen and Guan^20^ and Li & Zeng^54^, we defined the East Asian monsoon (EAM) by determining the monthly meridional wind anomalies over 110–140°E, 10–40°N. According to Hu and Fedorov^30^, Seiki and Takayabu^10^, and Chiang and Friedman^36^, cross-equatorial flow (CEF) plays an important role in the development of ENSO in the region of Papua New Guinea, where the MJO extends northward, from 120 to 150°E. Since this study examines the influence of the Australian subtropical ridge on westerly wind anomalies in the western Pacific region, we defined the CEF as the monthly meridional wind anomalies over 140–170°E, 5°S–20°S.

### Australian subtropical ridge intensity (STR-I) index and position (STR-P) index

We utilise time series of the STR-I and STR-P following the definitions of previous studies^37–40^. We interpolate the zonal mean SLP to 0.5° resolution, using bilinear interpolation (CDO version 1.9.8^55^) and detect the location and pressure of the maximum values in the area 5–65°S, 147.5–152.5°E. The STR-I is the maximum zonally averaged SLP, whereas the STR-P describes the latitude where the maximum occurs. Note that the latitude is expressed in terms of degrees south (a higher positive value indicates higher latitude).

### Indian Ocean dipole mode index and Madden–Julian oscillation (MJO)

The Indian Ocean dipole mode is defined by the dipole mode index^56^, where the western pole covers 50–70°E and 10–10°N, and the eastern pole covers 90–110°E and 10–0°S. A positive IOD period is characterised by the presence of cooler than average water in the tropical eastern Indian Ocean and warmer than average water in the tropical western Indian Ocean. Conversely, a negative IOD period is characterised by the presence of warmer than average water in the tropical eastern Indian Ocean and cooler than average water in the tropical western Indian Ocean. The MJO index is available from Bureau Meteorology of Australia (http://www.bom.gov.au/climate/mjo/).

### Ensemble empirical mode decomposition (EEMD)

The Hilbert–Huang transform (HHT)/empirical mode decomposition (EMD^35,46^) is a relatively new empirical technique for analysing non-stationary and nonlinear time series, and it has only recently been applied in climate research^35,47–50^. Compared with other methods, such as wavelet and spectral analysis, EMD is more intuitive, direct, and adaptable^51^. Through this method, the original signal can be adaptively decomposed into a series of independent modes, called intrinsic mode functions (IMFs), which have different dominant frequencies. Since the decomposition acts on the inherent nature of the original signal, EMD is a self-adaptive signal processing method. EMD assumes that different oscillatory modes constitute a given dataset, each with individual physical meanings^44^, with their frequencies and amplitudes classifying each empirical mode. Unlike canonical Fourier transform (FT) analysis, frequencies and amplitudes in EMD are functions of time.

In EMD analysis, the data are initially decomposed into a set of IMFs, as shown in Figure S3. The decomposition process is called a sifting process and consists of three main steps: (1) identify the maxima and minima in the input data; (2) form upper and lower envelopes by fitting the extremes with cubic splines; and (3) calculate the differences between the envelope functions and the mean to provide an estimate of the first component h1. This first component, *h1*, is then treated the same as the original data and the steps are repeated to obtain improved estimates of the first intrinsic mode function (IMF_1_). IMFs obey the following conditions: (a) in the whole data domain, the number of extrema and the number of zero crossings is either equal or differs at most by one; (b) at any point, the mean value of the envelope is zero. The residual of x (t) and IMF_1_ is then taken as a new time series r_1_(t), and a second sifting process is applied. The sifting process is completed when the residual r_n_(t) becomes a monotonic function. The time series x(t) can be decomposed as follows:

1 $$x\left(t\right)={IMF}_{1}+{r}_{1}(\mathrm{t}),$$

2 $${r}_{1}\left(t\right)={IMF}_{2}+{r}_{2}(\mathrm{t}),$$

3 $${r}_{n-1}\left(t\right)={IMF}_{n}+{r}_{n}(\mathrm{t}),$$

4 $$x\left(t\right)=\sum_{j=1}^{n}{IMF}_{j}+{r}_{n}(\mathrm{t}),$$

In this study, we primarily used ensemble empirical mode decomposition (EEMD^52^). EEMD is an improved version of EMD that was developed to resolve the problem of mode mixing (i.e., different time scales characterising the same IMF or similar timescales characterising different IMFs). For example, over the central Pacific, IMFs of SST anomalies contain both seasonal and annual signals^51,53^, which would normally be expected to separate into two different modes. The only difference between EMD and EEMD is that in EEMD, white noise is added to the data prior to performing the EMD. The main parameter settings for EEMD are the number of replication members that are to be used and the standard deviation of the Gaussian random number, which is described by the signal-to-noise ratio (SNR). Because the number of replication members constitutes the number of ensemble replicas over which the averaging process performed, this number must be high enough to provide the averaging process with a statistical meaning, but not low enough to maintain computational feasibility. We selected 200 as a reasonable compromise between these two requirements and set the SNR to 0.5 as this is the minimum value to prevent mode mixing (note that different samples become indistinguishable as SNR goes to zero). For more details on EEMD, please refer to NCL, the NCAR command language software (NCL; https://www.ncl.ucar.edu/), version 6.6.2^57^.

## Supplementary Information

Supplementary Information.

## Data Availability

The data supporting the findings of this study are available within the article. Any other data are available from the corresponding author upon request.
