# Supplementary material for: Generation of westerly wind bursts by forcing outside the tropics
Source: Sci Rep. 2021 Jan 13;11:912. doi: 10.1038/s41598-020-79655-7 (PMC7806706; doi:10.1038/s41598-020-79655-7)
Supplement: Supplementary file 1 — Supplementary Information. [file 41598_2020_79655_MOESM1_ESM.pdf]

Generation of westerly wind bursts by forcing outside the tropics

Supplementary information

Arnold Sullivan[1,2]; Wenxiu Zhong[3,4]; Gian Luca Eusebi Borzelli[5]; Tao Geng[6,7]; Chloe Mackallah[1]; Benjamin Ng[1]; Chi-Cherng Hong[8]; Wenju Cai[1,6,7]; An-Yi Huang[8]; Roger Bodman[1,9]

[1]CSIRO Oceans and Atmosphere, Aspendale, Australia;
[2]School of Earth, Atmosphere, and Environment, Monash University, Melbourne, Australia;
[3]School of Atmospheric Sciences, and Guangdong Province Key Laboratory for Climate Change and Natural Disaster Studies, Sun Yat-sen University, Guangzhou, China;
[4]Southern Laboratory of Ocean Science and Engineering (Guangdong, Zhuhai), Zhuhai, China;
[5]CERSE-Center for Remote Sensing of the Earth, Rome, Italy;
[6]Centre for Southern Hemisphere Oceans Research (CSHOR), CSIRO Oceans and Atmosphere, Hobart, Australia;
[7]Key Laboratory of Physical Oceanography/Institute for Advanced Ocean Studies, Ocean University of China and Qingdao National Laboratory for Marine Science and Technology, Qingdao, China;
[8]Department of Earth and Life, University of Taipei, Taipei, ROC;
[9]School of Earth Sciences, University of Melbourne, Melbourne, Australia.

Correspondence: Arnold Sullivan (arnold.sullivan@csiro.au)

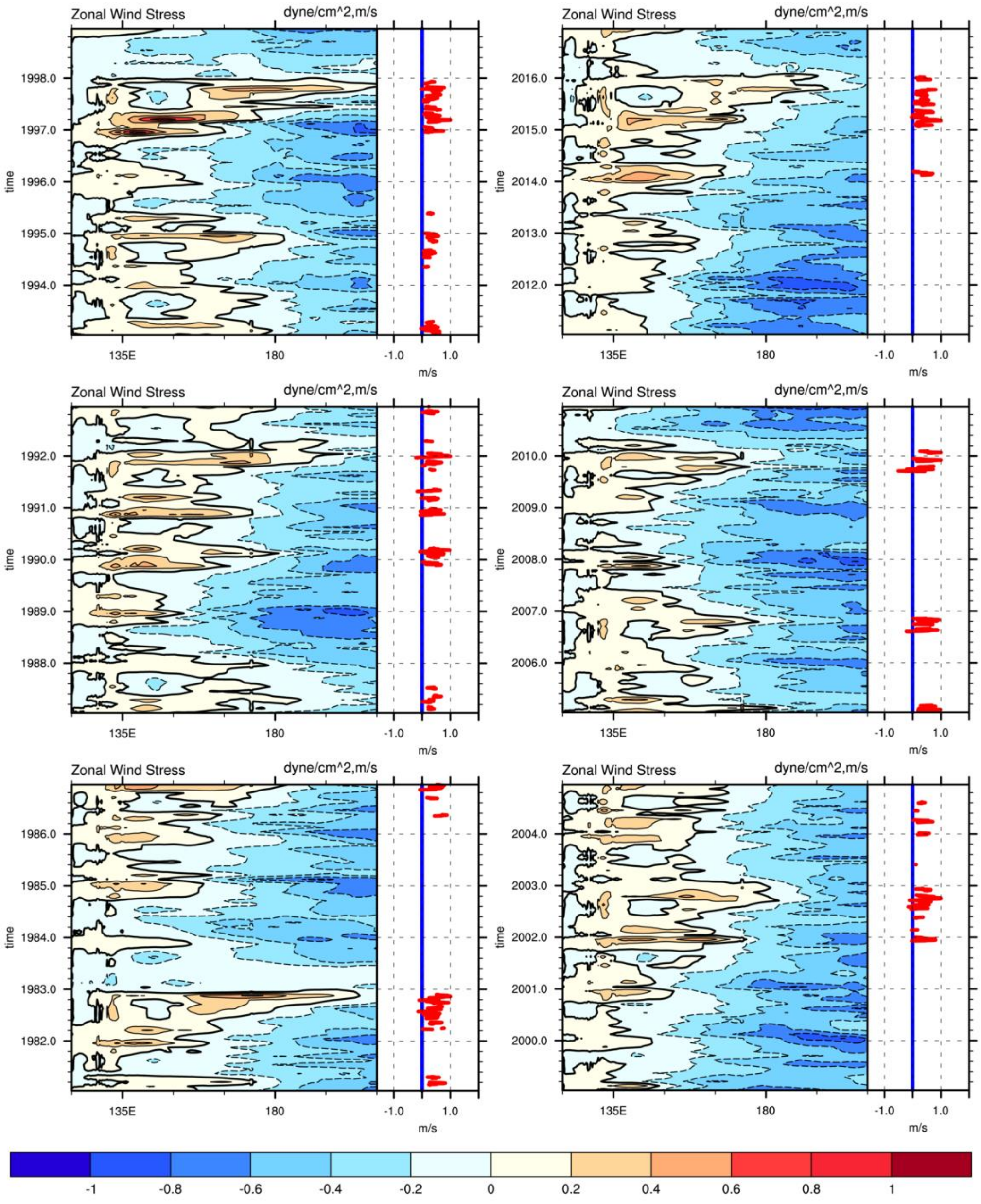

Figure S1. Hovmöller diagrams (longitude-time) of the monthly zonal wind stress averaged for 2ºS-2ºN (left panel; in dyne/cm2) interpolated daily. Solid contours are positive values and represent westerly wind; dashed contours represent easterly wind. Right panels are the WWBs divination obtained using the method proposed by Santoso et al., (2017) in red bars (units: m/s). Note that red curves follow the strong case when the orange shade in peaks of the zonal wind stress on the right. Maps were generated using the NCAR Command Language (NCL; https://www.ncl.ucar.edu/), version 6.6.2.

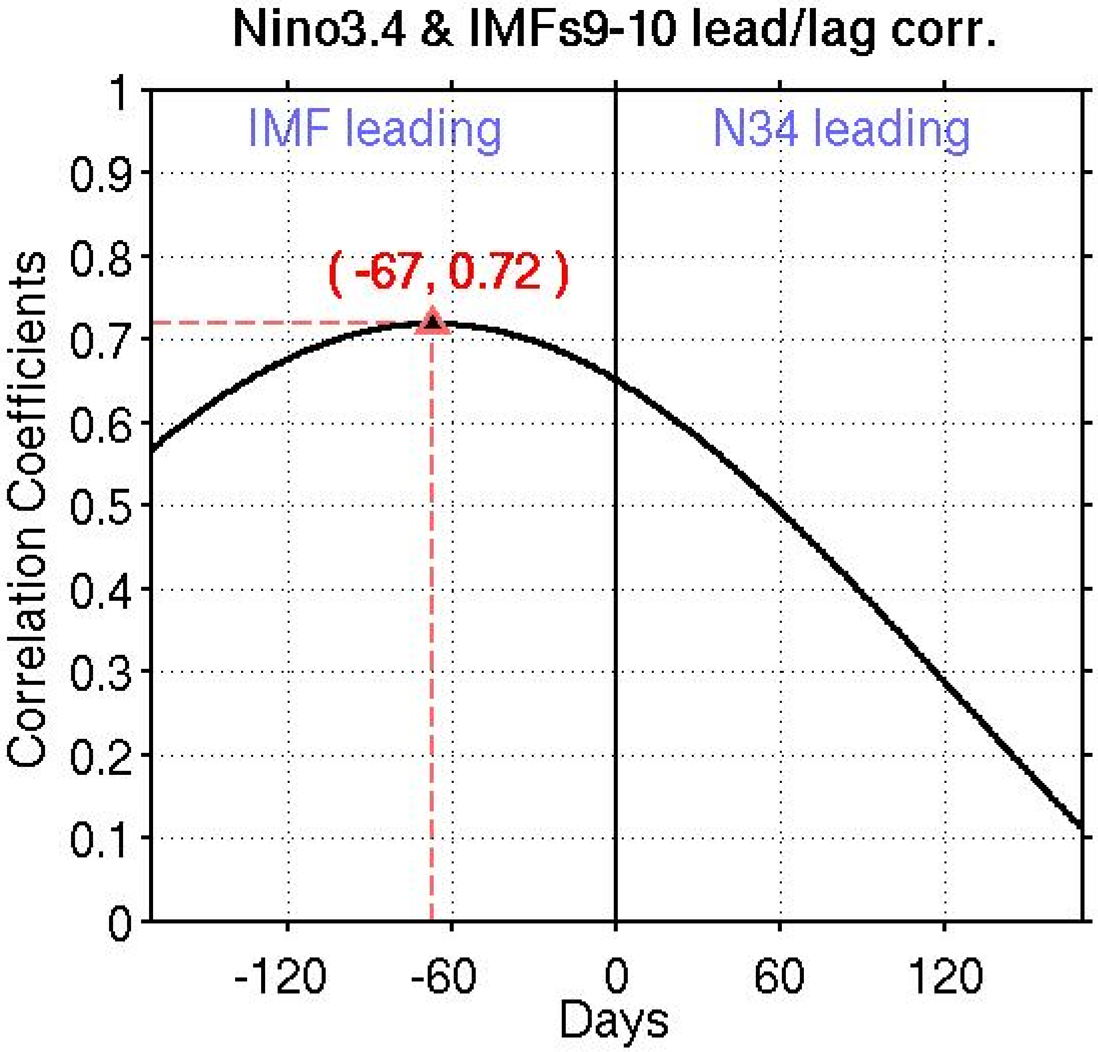

Figure S2. The lead and lag correlation coefficient plot between daily indices of Niño3.4 and IMFs 9-10. Data used cover the period 1981/9/1-2019/4/26. The negative part of the figure shows that the IMFs 9-10 leads Niño3.4 of 67 days, and the maximum correlation between IMFs 9-10 and Niño3.4 is 0.72, which is statistically significant. This figure was created using Matlab (**MATLAB. version 7.10.0 (R2010a). Natick, Massachusetts: The MathWorks Inc.; 2010.**).

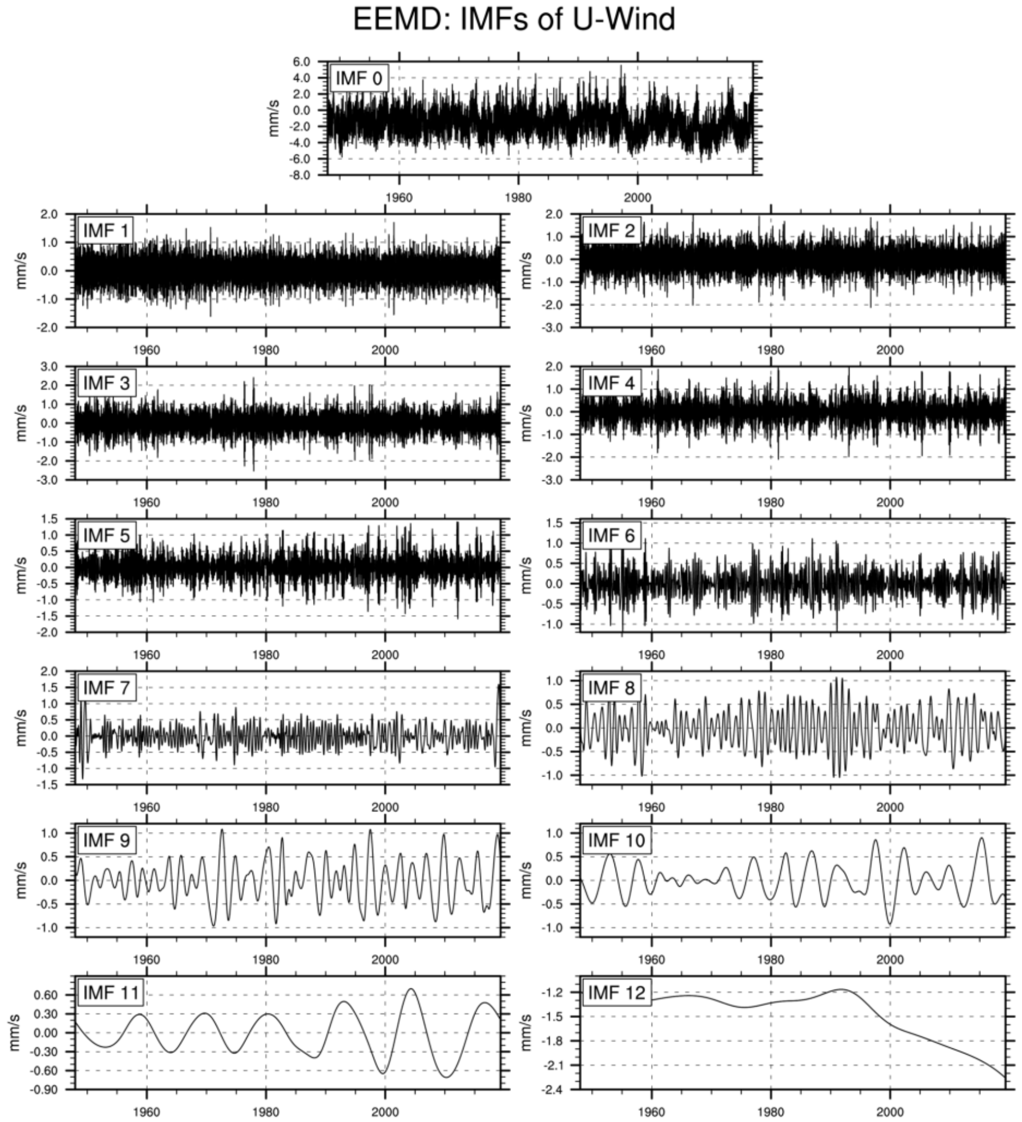

Figure S3. The plot is the decomposition of WWB using EEMD. IMF-0 is the raw daily WWBs. The variances for IMFs 1-5 are **0.131**, **0.199**, **0.251**, **0.242**, **0.147**, for IMFs 6-10 are **0.109, 0.094, 0.149, 0.183, 0.123**, and for IMFs 11-12 are **0.108, 0.065**. The total variance of the raw westerly wind event is **1.80**. IMFs 1-5 explained 53% of the noise part of the WWBs, and IMFs 6-8 explained 20% of the WWBs. The residual contained interannual and decadal signal and that took 27% of the WWBs.

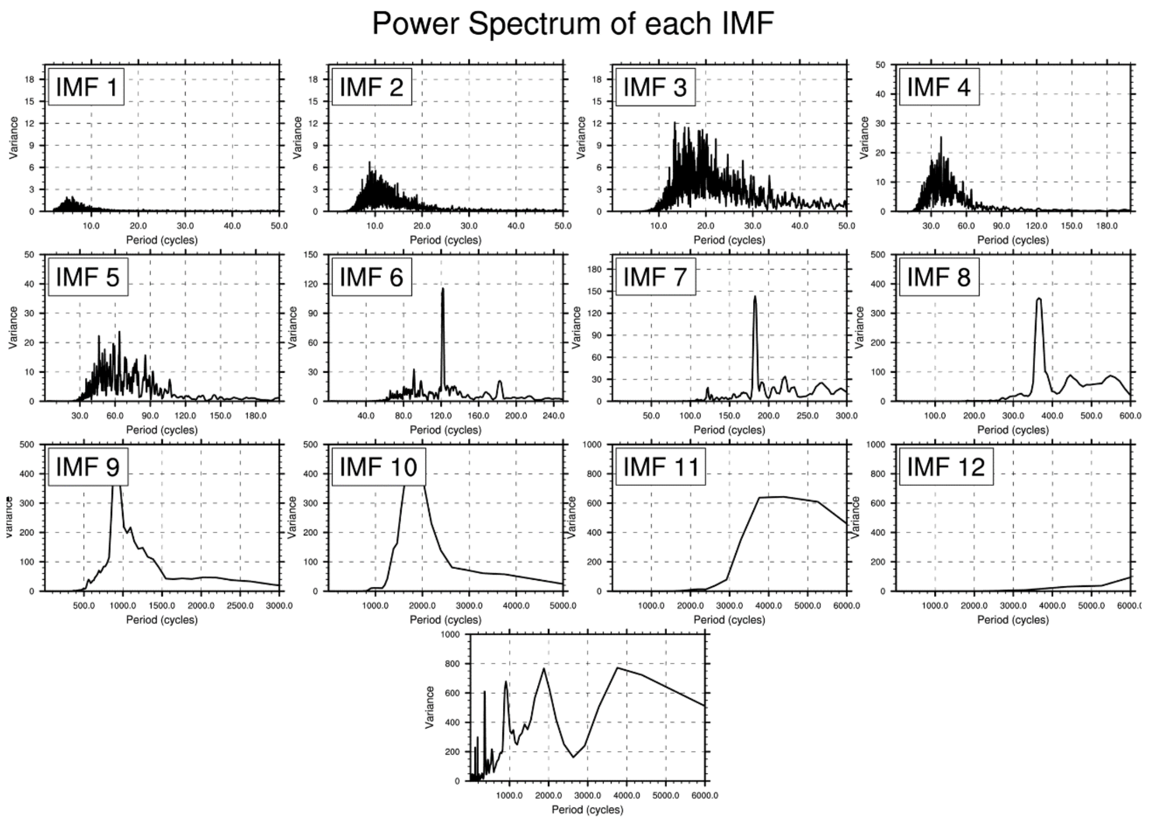

Figure S4. Fast Fourier Transforms (FFT) Power Spectrum of IMFs and the power spectrum of WWBs (bottom). Frequencies (i.e. the x-axis) is in days^{-1}. The power spectra of IMFs 1-5 show no significant peaks; a discussion on this result is provided in the following section. Power spectra of IMF6, IMF7 and IMF8 show clear peaks at 120days, 180days and 360days. IMF9 and IMF10 are ENSO related signals. IMFs 11-12 are the multi-decadal and trend. The bottom plot is the power spectrum of the raw westerly wind over the western Pacific region (5°S~5°N, 135°E~180°E). The bottom plot is the spectrum of the origin WWBs and shows the power spectrum peaks at 120 days, 365days and other interannual and longer time domain.

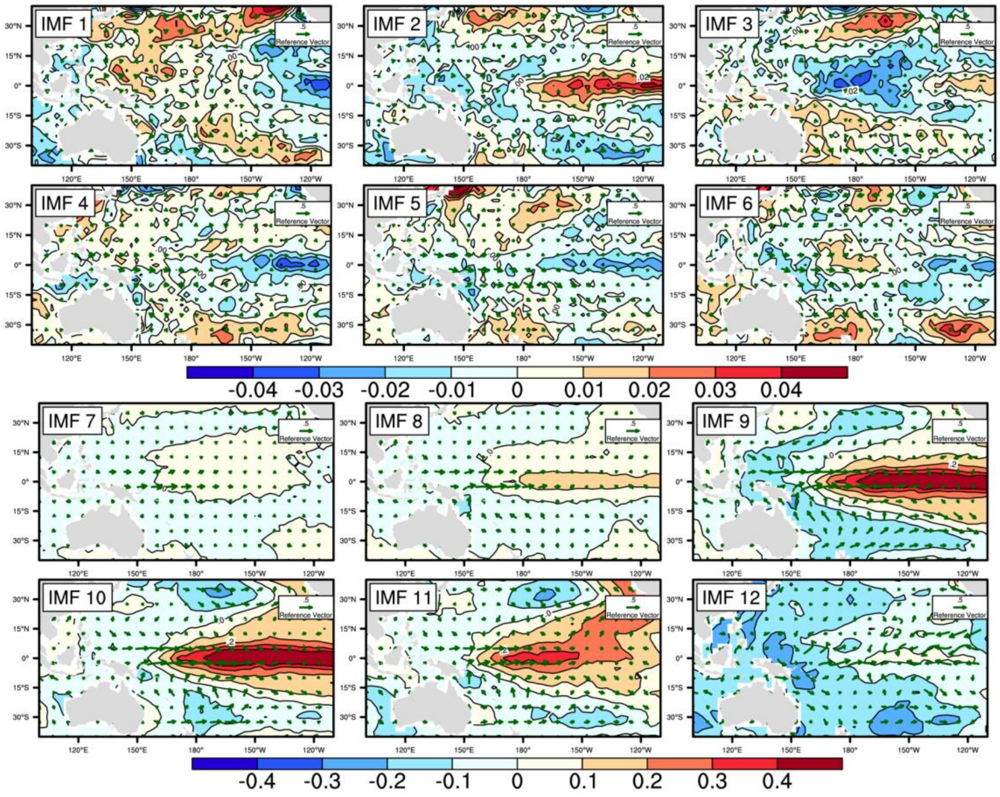

Figure S5. Regression coefficient patterns of IMFs onto the SSTa and the wind anomalies. Top panels are the IMFs 1-6 with SSTa (contour interval of 0.02 C^{o} between -0.04 C^{o} and 0.04 C^{o}. Bottom panels are the IMFs 7-12 with contour intervals of 0.1 C^{o} in the range -0.4 C^{o} - 0.4 C^{o}. Maps were generated using the NCAR Command Language (NCL; https://www.ncl.ucar.edu/), version 6.6.2.

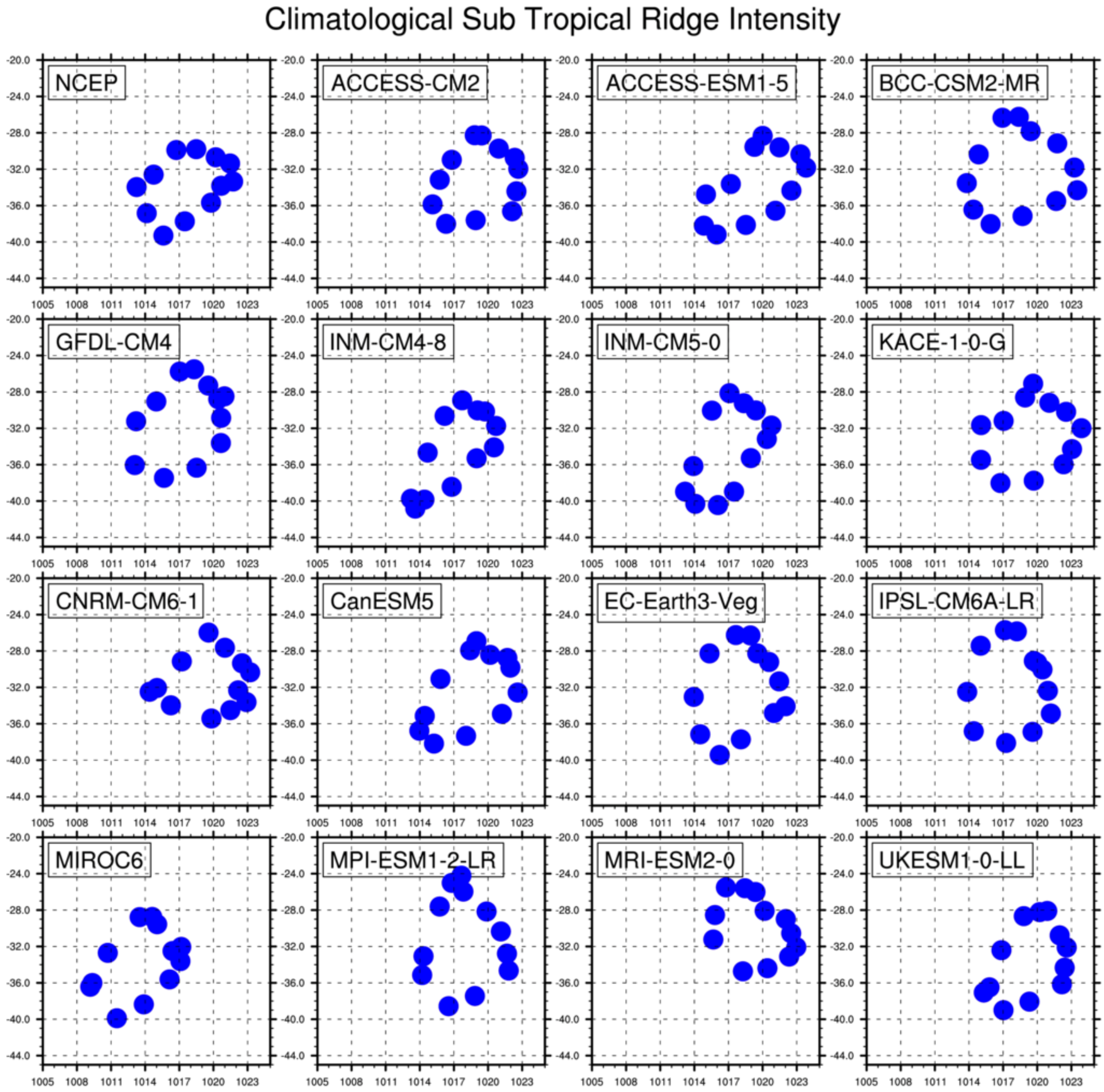

Figure S6. Annual cycle of the STR-P (y-axis, degrees latitude) and STR-I (x-axis, hPa) in NCEP and 15 CMIP6 models (red: historical 1950-2014; green: SSP585 2051:2010). The indices show that the maximum intensity and northward extent occur from June to August (toward the right top) and the minimum intensity takes place between January and February (toward the left bottom). Heading toward the future climate, not only the intensity of the STR increases, but the centre of the STR reallocates southward too.

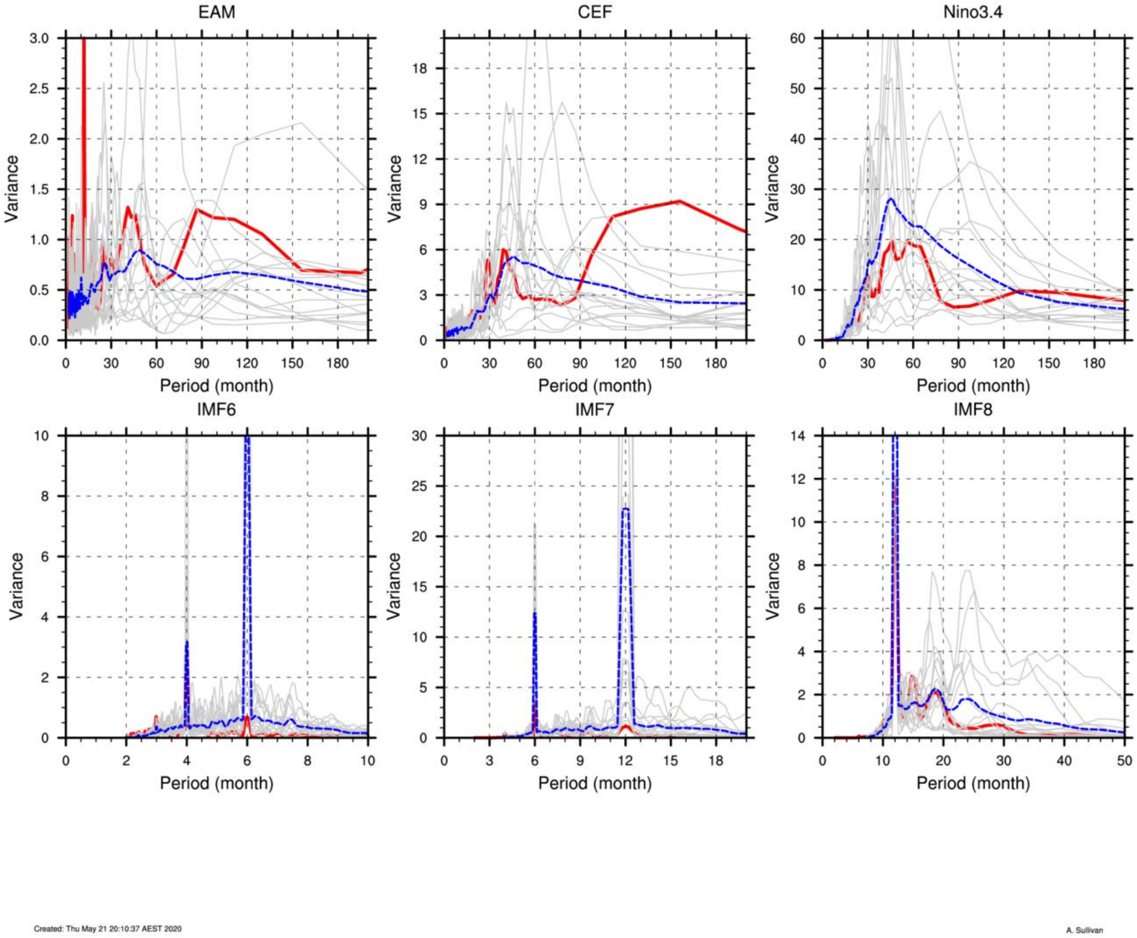

Figure S7. Plots of EAM, CEF, Niño3.4 and IMFs 6-8 power spectra. The grey curves indicate modes in the historical scenarios, and the blue curves are the model ensemble means. The red curves are the results from the reanalysis. Models are in a wide range of power spectrum in the simulation in EAS, CEF and particularly the ENSO simulation. From the decomposition of the westerly wind, most of the models can catch the right peaks such as four months and six months in IMF6, six months and 12 months in IMF7, 12 months in IMF8. The variance from the models are stronger than reanalysis.

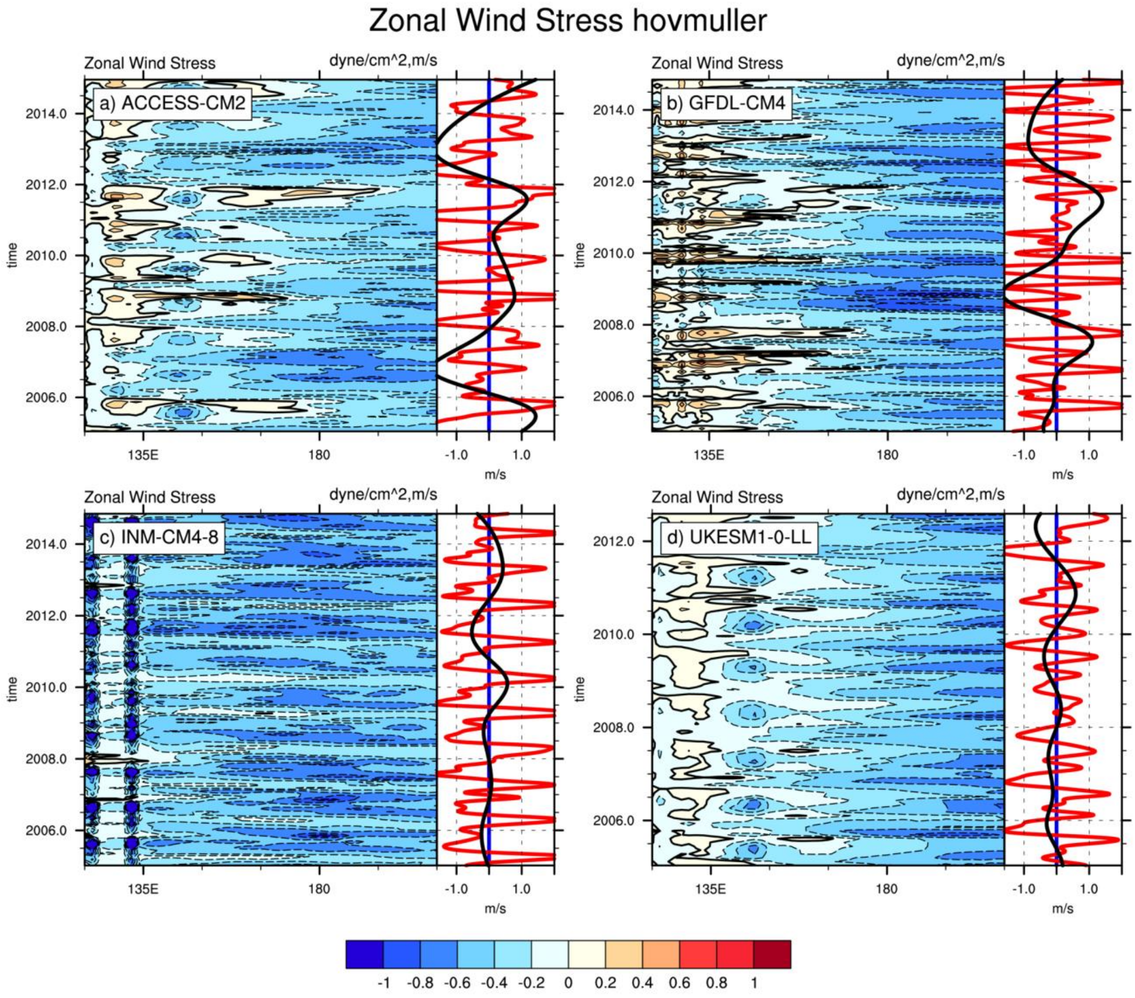

Figure S8. The time-longitude contour plot of the zonal wind stress along the Pacific equator (left panels; in dyne/cm2) and the IMFs 6-8 in red, and the IMFs 9-10 in black curves (right panels; in m/s). The last ten years data were used depends on the data availability. **a** Strong easterly wind is present in ACCESS-CM2, and the zero line of the zonal wind stress almost reaches to the 120°E. Only during the El Nino event, the strong westerly zonal wind stress can be found to cross the 150°E. Meanwhile, the intra-seasonal high-frequency westerly wind can be found along the 135°E, which are followed by the red curve on the right-hand side. **b** Similar to the observation, the zero zonal wind is clearly observed from the black curve on the right-hand side from the GFDL-CM4. **c** INM-CM4-8 has very weak El Nino, and the variance of the IMFs 9-10 is very small, unlike the IMFs 6-8. From the zonal wind stress evolution pattern, the westerly wind can barely be found. However, the seasonal fluctuation of the IMFs 6-8 is still strong. It is interested to have further investigation over the relationship between westerly wind anomalies and the development of the El Nino in this model. **d** The evolution of the zonal wind stress and the decomposition of the westerly wind from the UKESM1-0-LL are comparable to the ACCESS-CM2 with the same version of the atmospheric model. Maps were generated using the NCAR Command Language (NCL; https://www.ncl.ucar.edu/), version 6.6.2.

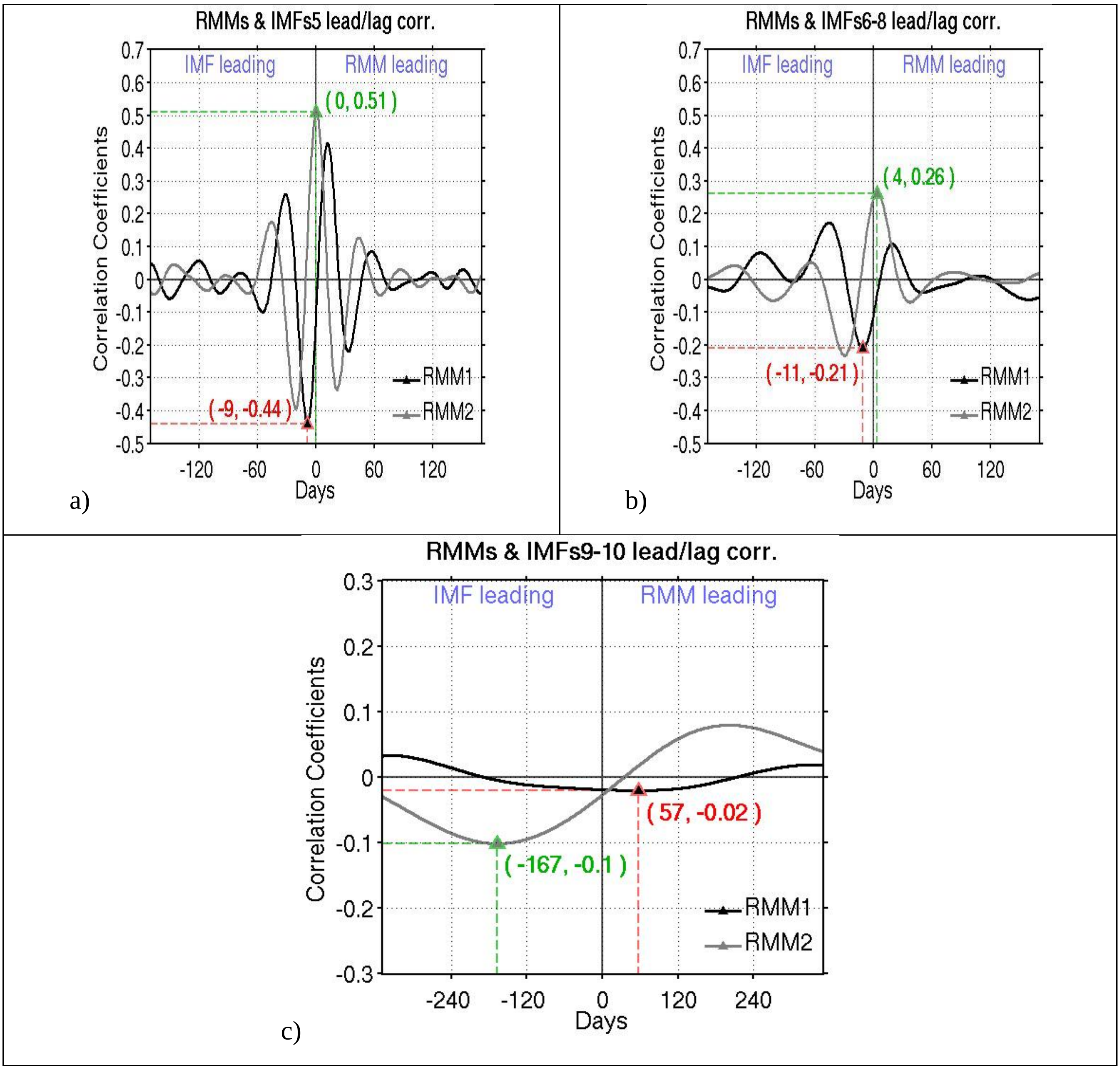

Figure S9. Lead and lag correlation coefficient between: a) Lead and lag correlation coefficient between IMF5 and MJO, RMM1 and RMM2. IMF5 leads RMM1 of nine days, and the corresponding correlation is -0.44, where it is 0.51 between IMF5 and RMM2 at zero lag. b) Lead and lag correlation between MJO indices and IMFs 6-8. There is no significant leading period between MJO and IMF5 or IMFs 6-8, and both are less 20 days. c) is the relationship with IMFs 9-10, the ENSO related wind, which is leading MJO RMM2 in 167 days and RMM2 is leading IMFs 9-10 57 days, however the coefficient is comparatively small. The software used to generate the lead-lag plots in Matlab (**MATLAB. version 7.10.0 (R2010a). Natick, Massachusetts: The MathWorks Inc.; 2010.**).

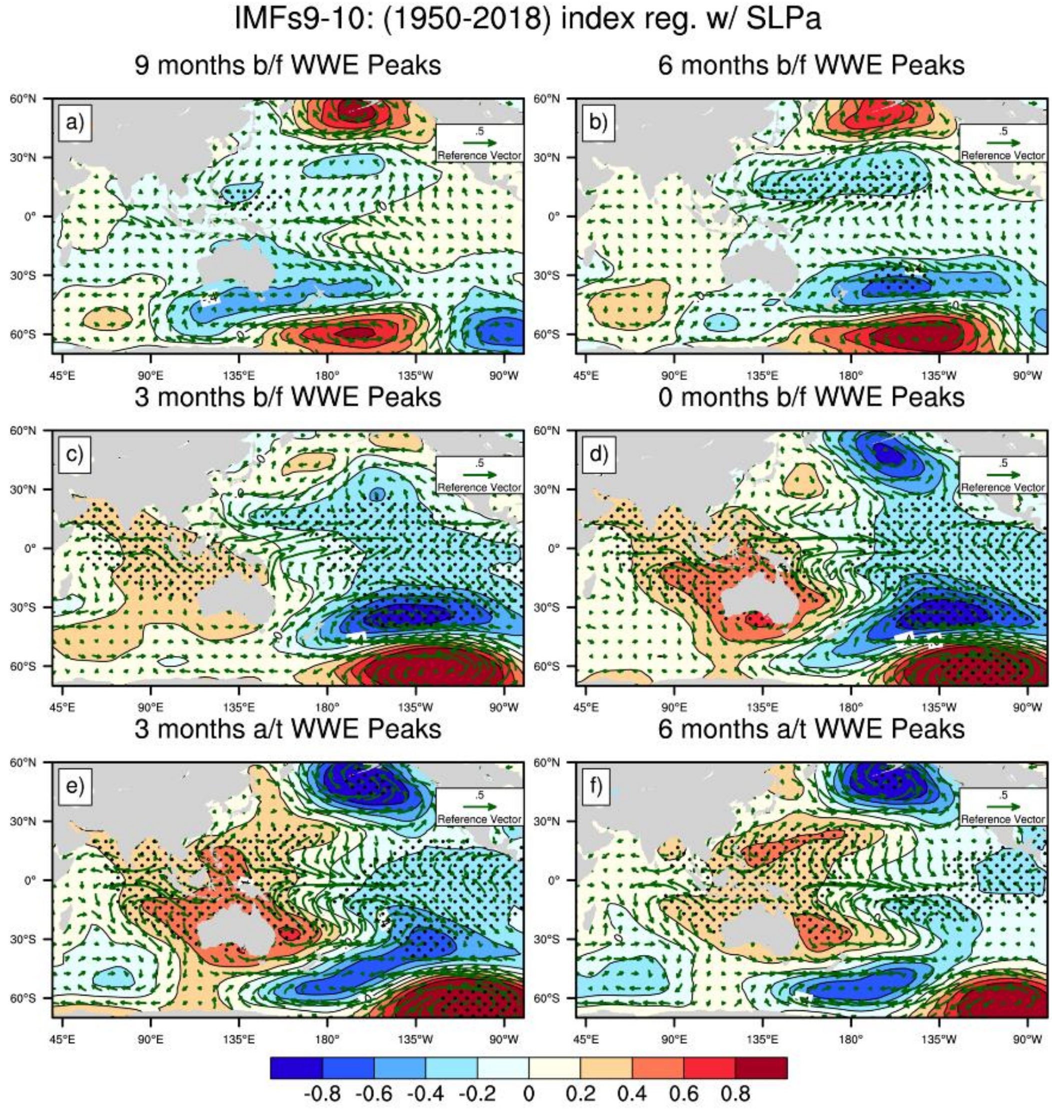

Figure S10. Regression patterns between monthly means of IMFs 9-10 and sea level pressure anomaly. Maps were generated using the NCAR Command Language (NCL; https://www.ncl.ucar.edu/), version 6.6.2.

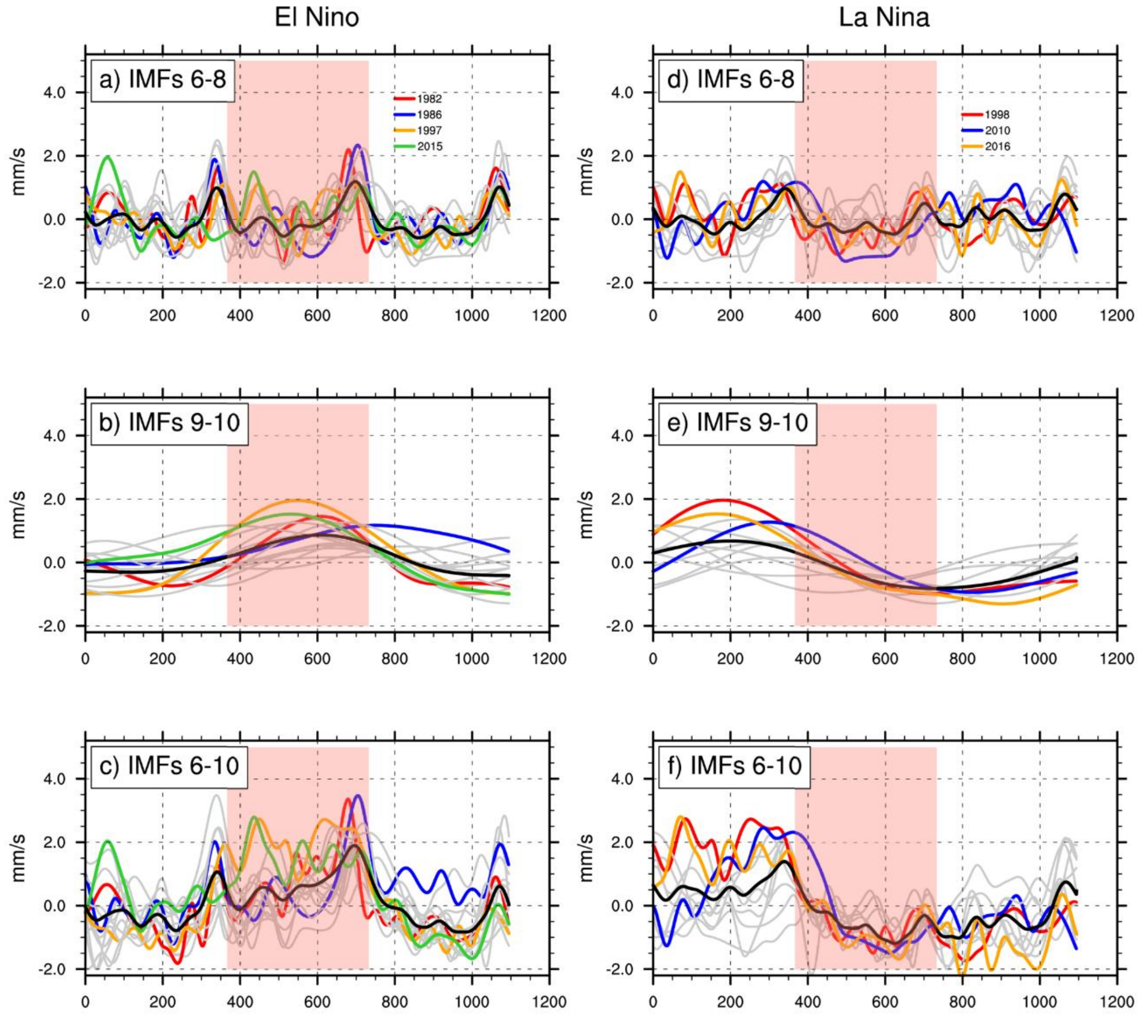

Figure S11. a) Evolution of the IMFs 6-8 decomposed from the daily zonal wind in El Niño years, covering from one year before (start from day1) to one year after (end at day 1095) the El Niño peak year, which is in the shade in salmon colour. Coloured curves represent strong El Niño cases of year 1982, 1986, 1997 and 2015, and grey curves indicate other moderate El Niño years. The ensemble means of IMFs 6-8 for all El Niño cases is shown in thick black curve. b-c) are the same to a) but for IMFs 9-10 and IMFs 6-10 in El Niño events. d-f) are the same to a-c) but for La Niña years.

Table S1

	DJF					MAM				
	corr(IMF,EAM)	corr(IMF,CEF)	corr(IMF,STR-I)	corr(CEF,EAM)	corr(CEF,STR-I)	corr(IMF,EAM)	corr(IMF,CEF)	corr(IMF,STR-I)	corr(CEF,EAM)	corr(CEF,STR-I)
**NCEP**	0.29	0.34		0.37	0.29	-0.41				0.37
**ACCESS-CM2**	0.34	0.55	-0.23	0.42		-0.22		0.22		0.36
**ACCESS-ESM1-5**		0.42				-0.33			0.21	
**BCC-CSM2-MR**	0.25	0.52	0.26	0.33			-0.26			
**GFDL-CM4**	0.42	0.56		0.39	0.29	-0.33		-0.21	0.44	0.49
**INM-CM4-8**				0.27	0.25		0.41			
**INM-CM5-0**							0.38		0.36	0.3
**KACE-1-0-G**	0.38	0.55		0.4	0.23	-0.28			0.3	0.51
**CNRM-CM6-1**				0.49	0.22					0.41
**CanESM5**	0.21		-0.4		-0.21	-0.23	-0.49		0.3	0.46
**EC-Earth3-Veg**	0.34	0.47		0.46	0.25				0.31	0.38
**IPSL-CM6A-LR**	0.42						-0.67	-0.21		
**MIROC6**	0.38	0.44		0.58		-0.52	-0.37	-0.24	0.46	0.48
**MPI-ESM1-2-LR**				0.36			0.32		0.45	0.42
**MRI-ESM2-0**	0.41	0.61		0.47	0.27	-0.5	-0.23	-0.29	0.33	0.44
**UKESM1-0-LL**	0.42	0.43		0.28		-0.5	-0.41		0.55	0.22

	JJA					SON				
	corr(IMF,EAM)	corr(IMF,CEF)	corr(IMF,STR-I)	corr(CEF,EAM)	corr(CEF,STR-I)	corr(IMF,EAM)	corr(IMF,CEF)	corr(IMF,STR-I)	corr(CEF,EAM)	corr(CEF,STR-I)
**NCEP**	0.24				0.64		0.46		-0.33	0.65
**ACCESS-CM2**		0.56			0.3		0.52			0.62
**ACCESS-ESM1-5**		0.4		0.27	0.32		0.68			
**BCC-CSM2-MR**		0.24	0.35		0.55		0.35	0.32	-0.34	0.74
**GFDL-CM4**	-0.45	0.4		-0.5	0.74		0.54			0.73
**INM-CM4-8**	0.41				0.5			0.33		0.38
**INM-CM5-0**		0.35			0.36		0.38			0.58
**KACE-1-0-G**	-0.27	0.35			0.45		0.39			0.6
**CNRM-CM6-1**			0.26	0.33	0.53		0.52	0.24		0.75
**CanESM5**		0.33	0.35		0.56	-0.23	0.22	-0.25		0.47
**EC-Earth3-Veg**	-0.28				0.59	-0.26	0.3			0.75
**IPSL-CM6A-LR**					0.47		0.45			0.53
**MIROC6**		0.28		0.38	0.71		0.35	0.26		0.64
**MPI-ESM1-2-LR**					0.55	-0.4	0.38	0.25		0.72
**MRI-ESM2-0**		-0.43			0.46		0.47			0.73
**UKESM1-0-LL**	-0.24		0.26		0.62		0.49			0.45

The correlation coefficient between (IMFs 6-8, EAM), (IMFs 6-8, CEF), (IMFs 6-8, STR-I), (CEF, EAM), (CEF, STR-I) in **15** CMIP6 models are present and also breaks into four different seasons, DJF, MAMA, JJA and SON. All the correlation coefficients pass the 99% statistically significant level are shown. The spreadsheet was created by Microsoft Excel.

Supplementary Information of EEMD/HHSA:

The method introduced in this study to deal with Westerly Wind Bursts (WWBs) is different from the traditional approach, such as Fourier Transform (FT). This is because of the following facts: 1) the FT is inefficient in dealing with a nonlinear and nonstationary signal, 2) as WWBs last between 5 and 30 days, they cannot be considered simply as atmospheric noise. It is indeed our hypothesis that WWBs are the result of the modulation that atmospheric noise exerts on the amplitude of zonal winds in the tropical Pacific. To understand the concept of Amplitude Modulation (AM) in its basic form and defects of FT to deal with such modulated signals, consider, as in Figure SI1, for instance, a cosine wave with a frequency of 125Hz (this is an entirely natural frequency to test). Adding white noise to the original cosine signal, provided that noise amplitude remains not too high respect to the amplitude of the signal, FT is capable of capturing the signal embedded into the noisy pattern of the resulting signal. Consider, instead, the (nonlinear) case in which the cosine wave is multiplied by a white noise signal. This is precisely an example of amplitude modulation (i.e. the amplitude of the cosine wave is modulated by noise). Traditional FT is unable to deal with such a modulated signal. That's why, in this study, the new method EEMD/HHSA has been applied. Here we just discuss the basic concepts underlying EEMD/HHSA, for the details refer to Huang et al., (2016).

The amplitude modulation is an interaction between a high-frequency signal and a low-frequency signal in a multiplicative process. Figure SI1c shows the faulty of FT in dealing with such modulated signals. However, is FT, somehow, after some manipulation, capable of capturing the deterministic part in a signal modulated in this way? Note that the low-frequency signal, compared to noise, acts as a carrying signal. According to Huang et al. (2016), the carrying signal will be present on the amplitude of the AM signal. Here we simply square the modulated signal. As it is possible to see in Figure SI1d, F.T. can catch the signal, even if the frequency is doubled respect to the original owing to the fact that we flipped all negative phase to be positive, which imply double the frequency.

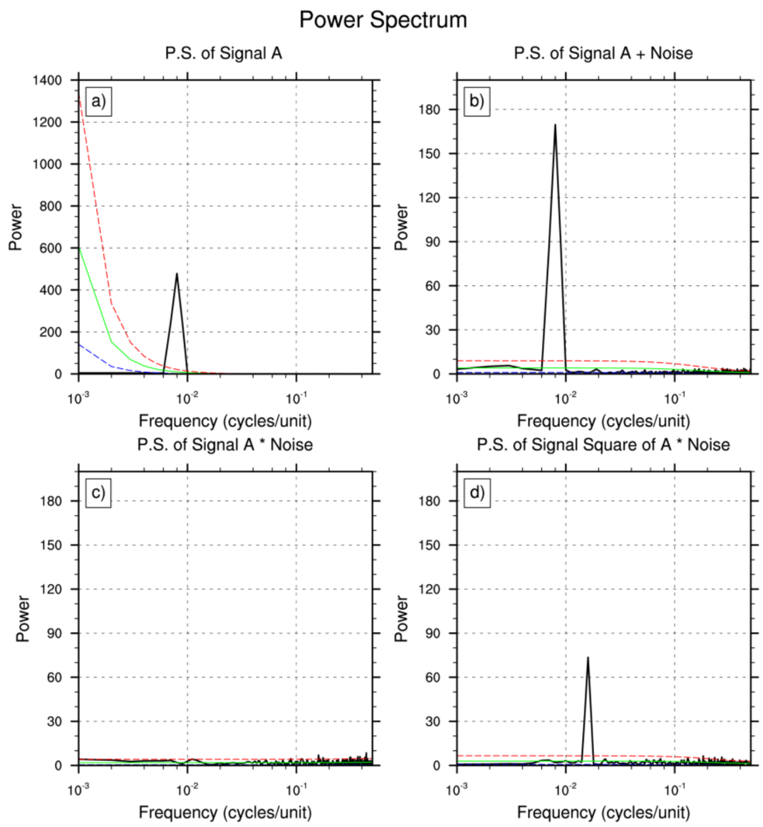

Figure SI1. A Fourier Transform power spectrum of a sine signal with frequency of 125Hz, A*cos(2*PI*frequency*ts) from zero to one second with sample rate 1000. **a,b** The power spectrum of the original signal and the signal added a white noise with one standard deviation.

c The power spectrum of the original signal multiplied by white noise. No significant peaks are shown in this panel. **d** The power spectrum of the squared signal via the multiplicative process, which shows no peak in panel **c**.

Why do we not include IMF5? Simply we square IMF5. Why we need to square the signal particularly in IMF5? In the previous session, we provide an example of a sinusoidal signal with a frequency of 125Hz. Figure SI1a shows the power spectrum from the FT and a clear peak at 125Hz. When the signal is via an additive process, the power of the spectrum becomes more significant as in Figure SI1b. However, if the signal is modulated through a multiplicative process, the resulting signal is still harmonic at higher frequency owing to the Product and Sum Formulas of sine and cosine waves, which is a basic concept of the FT The Product and Sum Formulas creates a significant problem in analysing nonlinear problems. Using the idea of HHSA, nonlinear modulation is simply due to the multiplicative process between a signal and noise. The carrying signal or the lower frequency is generally present in the amplitude or the envelopment function. We then square the signal, and then applied the FT Figure SI1d shows the peak located near 63Hz. Meaning by square the signal, we can get the carrying signal back. In here the original signal peak at 125Hz and how come here we got a half? It is merely due to the square. Once apply the square, such as a sine wave, the second half of the phase will be adjusted to positive so that the frequency will double, and the period of the square signal became half of the original. So that IMF5 is a modulated signal and FT cannot simply identify the main frequency. If we square the IMF5 then we can get the carrying signal out, and those carrying signals peak at mainly at the interannual time scale. However, the variation of those carrying signals is rarely weak compared to others, and such concepts can apply to IMFs 1-5.

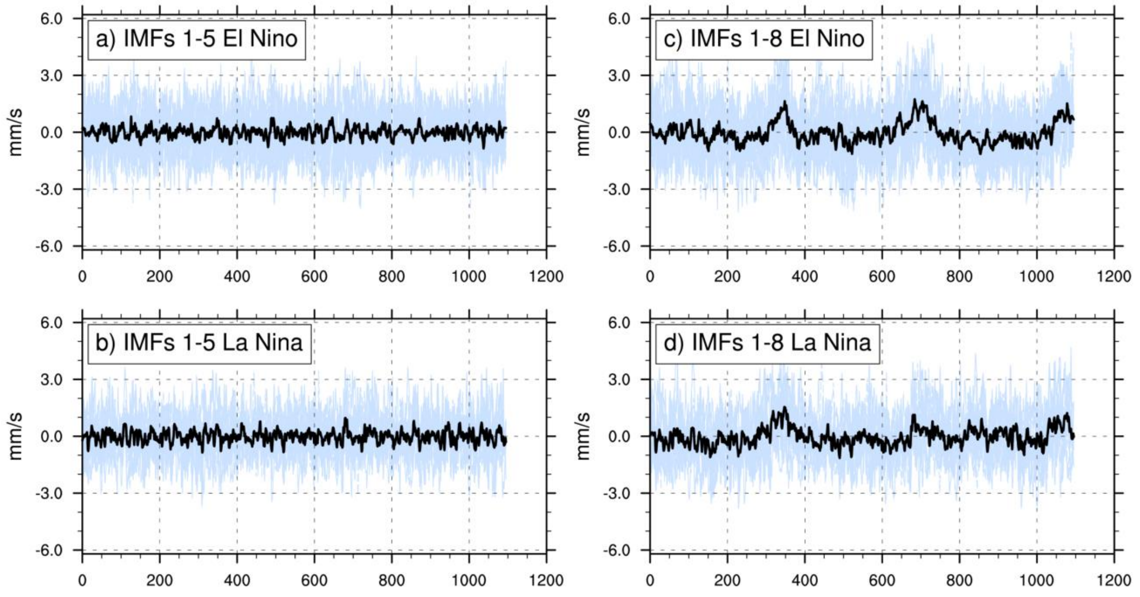

Figure SI2. A simple example is showing the modulation depth of the indices over the ENSO years, one year ahead and one year after in light blue curves. Ensemble means are in black curves. a Indices of IMFs 1-5 of one year before and one year after the El Niño years. **b** Over the La Niña years. **c,d** Same as **a,b** but only for IMFs 1-5. The plot shows the daily data; the x-axis is the time with units in days, starting from the 1 January a year before ENSO event till a year after ENSO. Due to the modulation of the intra-seasonal signal, the ensemble curves show the two peaks a year before ENSO and during ENSO year only when IMF6-8 are included; so, we need to consider IMFs 1-5 as not relevant to the generation of WWBs, which trigger ENSO events. More details on the concept of modulation depth will provide in the next section.

The carryings will be further weaker than the one on IMF5, so that we do not consider there is any significant signal about ENSO in IMFs 1-5 (Figure SI2). Previous studies argued that the WWB is noise; in Figure SI2a, b, again, we show, that by

accounting solely for IMFs 1-5, no wind burst can be observed during El Niño and La Nina: WWBs can be detected during these episodes only if IMF6-8 are included in the analysis (Figure SI2c and d).

The concept of the "modulation depth" of the amplitude modulation between a low-frequency and a noise

Modulation depth applied variously in recharge and discharge and recharge oscillator mechanism as the "noise amplitude". Here we try to explain what the primary role of the modulation depth in WWB is. In the available literature, WWBs are defined as westerly winds with intensity higher than 7 m/s and lasting from a few days to a month, but never more than a month. This definition appears misleading, when compared to another common idea, which relates WWBs to atmospheric noise. In order to clarify this issue, we introduced the concept of AM. In 2016, Holo–Hilbert spectral analysis (HHSA) extended the HHT/EEMD method to identify the amplitude-modulated (AM) characteristics of two signals through the nonlinear multiplicative process. HHSA indicates how the two signals can react through the nonlinear process and get involved with the cross-scale interaction of phase-amplitude modulation.

Finally, we explain that WWBs as intense as 7m/s must be attributed to the carrier signal and, in this context, we found that the seasonal monsoon from the northern and southern hemisphere plays an important role. Naturally, more the carrier signal is pronounced, more WWBs are strong. The enhancement of WWBs is not related to noise contribution, but to an increase of the carrier signal. We again use the concept of Holo-Hilbert spectral analysis and repeat the same demonstration of the amplitude modulation (AM). The sinusoidal wave here consists of a cosine wave with a frequency of 125 Hz and the exponential, $\cos(\Theta * t) * \exp(t)$, decays in time with very little e-folding time but a significant reduction in amplitude. This sinusoidal can be treated as the low-frequency signal, which, in this case, represents the intra-seasonal variability. Figure SI3b shows a damped oscillation with a frequency close to 125Hz, and, Figure SI3a, shows white noise with an amplitude equal to one standard deviation. Both signals have been reproduced for 1000 steps. Such white noise can represent as the atmospheric variability in this study. Here we focus on three concepts: the amplitude modulation, the modulation depth and the carrying signal, which is represented by the envelope function when modulated with other high-frequency signal or noise.

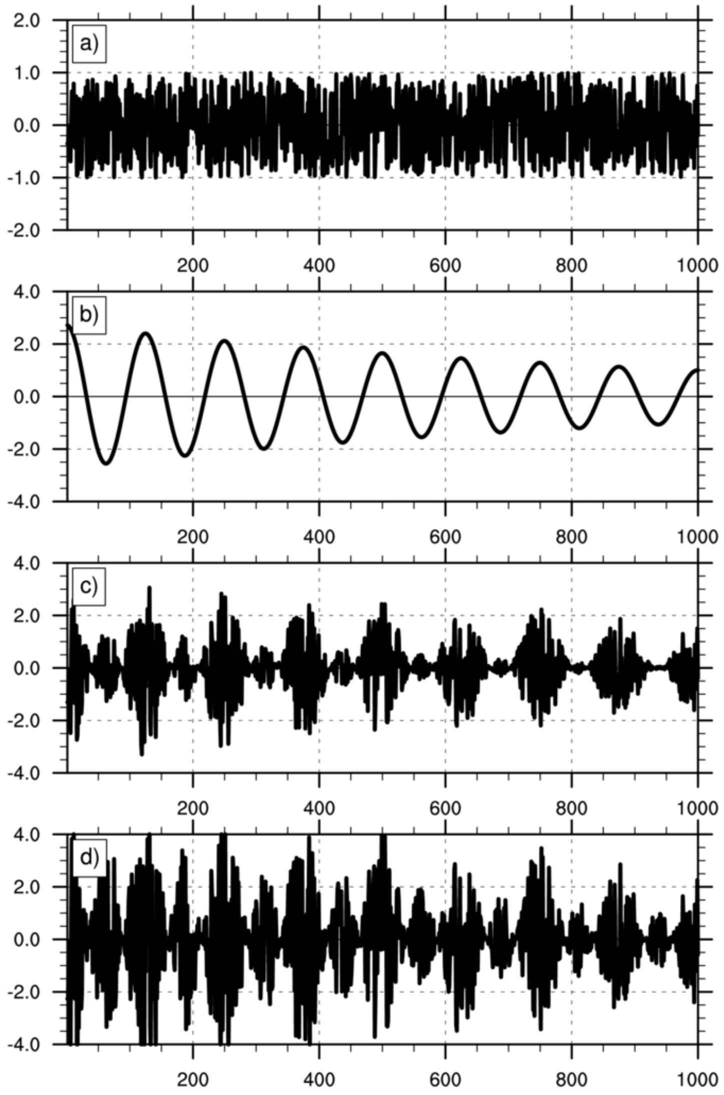

Figure SI3. a White noise with one standard deviation. **b** The chirp signal cos(Θ*t) * exp(t). **c**,**d** The product of **a** and **b,** but with different modulation depth.

Figures S15c, d show the products of white noise and the chirp signal with different modulation depth, one and two, respectively. The amplitude is higher in Figure SI3d, demonstrating that, although the input signals are the same, due to the different modulation depths, the amplitudes are different (e.q. Figure SI4). Modulation depth related to amplitude modulation between extratropical and other unknown signals, may explain why, at the western Pacific boundary, the recorded intensity of zonal wind anomaly is just 1 m/s, while over the interior of the basin, might be as high as seven m/s.

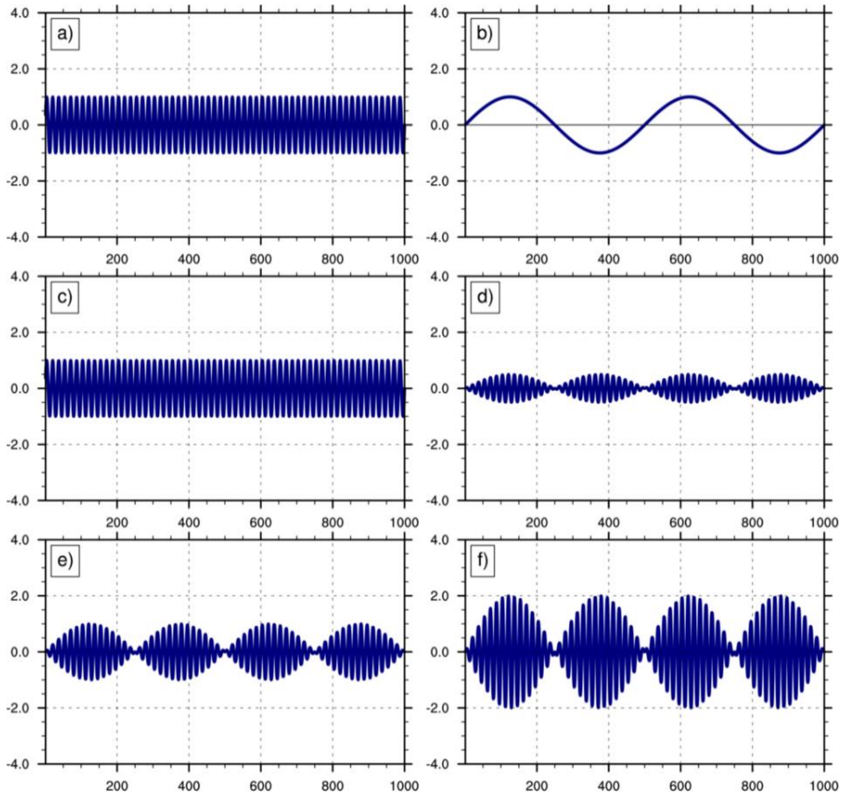

Figure SI4. Example of amplitude modulation of two signals. a sin(2*PI/50*3*ts) and b) sin(2*PI/50/10*ts) to represent a high and a low-frequency signal. **c-f** are the products from **a** multiplied by **b**, but with different modulation depth (MD) which is sin(2*PI/50*3*ts) * (MD/100)*sin(2*PI/50/10*ts), with MD from 0, 50, 100 and 200 respectively. **c-f** show the greater the MD, the greater the amplitude; however, the amplitude for both signals remain the same as one magnitude. So that the high frequency can nonlinearly modulated by amplitude modulation and the amplitude varies.

Finally, how can we identify the frequency of the multiplicative signal from traditional spectrum analysis? Figure SI5a, b shows the variance spectra obtained with different modulation depth from FT spectrum analysis. No trace of the sinusoidal wave, which should be around 125Hz, can be found in the resulting spectrum, and the peak frequency in Figure SI5a, b is not from neither chirp signal nor white noise. The other deficiency of the FT is that, in such a chirped signal, the frequency varies in time, which cannot be captured by FT analysis. But after square the signals, FT analysis shows 125Hz signal in Figure SI5c, and an extra harmonic signal, 63Hz signal in Figures S17d, due to product-to-sum formula. Preliminary investigations suggest that this high-frequency signal, such as atmospheric signal, is noise-like and modulated by the seasonal and annual cycle. The product of these two signals cannot be analysed correctly by using FT. HHSA is an indication of cross-scale coupling of phase-amplitude. Furthermore, this westerly wind event is most likely the high-frequency atmospheric signal modulated with signals from different timescale from seasonal to decadal. To investigate the composition of the westerly wind at the western Pacific is the leading study in this paper.

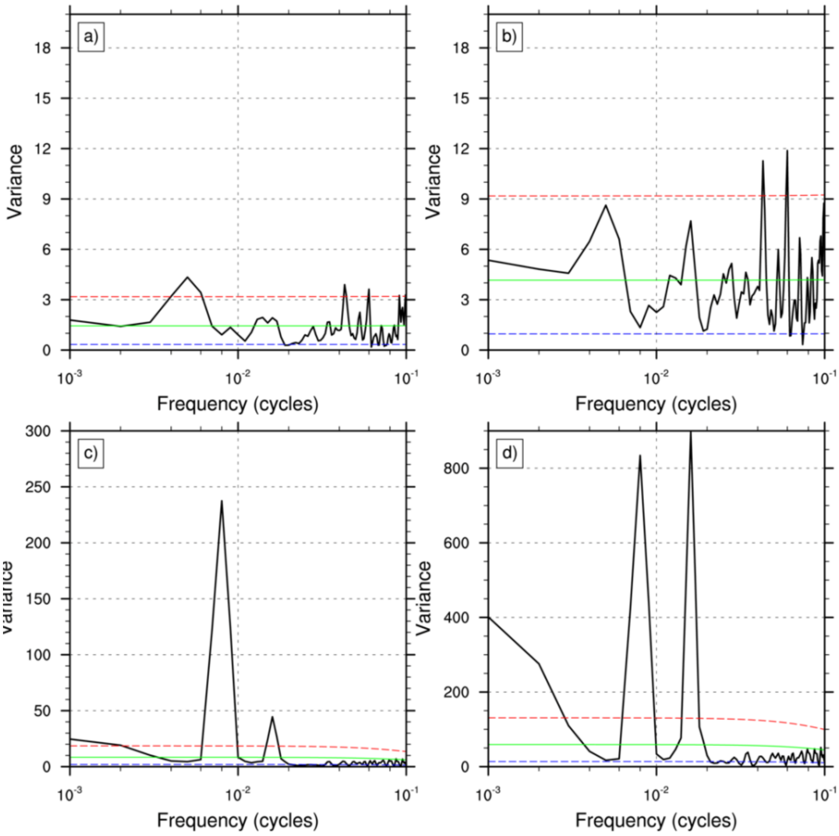

Figure SI5. Fourier spectrum analysis from the product of the chirp and white noise in Figure SI3c with a modulation depth of one **a** and two **b**. The red curves represent the red noise spectrum, and the green and blue curves are the upper and lower confidence bound. **c** and **d** are the power spectra of the squared signal of **a** and **b**. It is clear to see that the original frequency is around 125Hz and after the square, our results show the rate is near **63Hz** in **c** and both **125Hz** and **63Hz** finds in **d**.
